# Proteomic Analyses Reveal a Role of Cytoplasmic Droplets as an Energy Source during Epididymal Sperm Maturation 

**DOI:** 10.1371/journal.pone.0077466

**Published:** 2013-10-14

**Authors:** Shuiqiao Yuan, Huili Zheng, Zhihong Zheng, Wei Yan

**Affiliations:** 1 Department of Laboratory Animal Medicine, China Medical University, Shenyang, China; 2 Department of Physiology and Cell Biology, University of Nevada School of Medicine, Reno, Nevada, United States of America; University of Pecs Medical School, Hungary

## Abstract

A small portion of cytoplasm is generally retained as the cytoplasmic droplet (CD) on the flagellum of spermatozoa after spermiation in mice. CDs are believed to play a role in osmoadaptation by allowing water entrance or exit. However, many lines of evidence suggest that CDs may have roles beyond osmoregulation. To gain more insights, we purified CDs from murine epididymal spermatozoa and conducted proteomic analyses on proteins highly enriched in CDs. Among 105 proteins identified, 71 (68%) were enzymes involved in energy metabolism. We also found that sperm mitochondria underwent a reactivation process and glycolytic enzymes were further distributed and incorporated into different regions of the flagellum during epididymal sperm maturation. Both processes appeared to require CDs. Our data suggest that the CD represents a transient organelle that serves as an energy source essential for epididymal sperm maturation.

## Introduction

The release of fully developed spermatozoa from the seminiferous epithelium to the lumen of seminiferous tubules is achieved through a process termed spermiation [1]. During spermiation, the cytoplasm of spermatids is removed and phagocytosisized by Sertoli cells [2,3]. However, a small portion of the cytoplasm is usually retained and attached mostly to the middle piece of the flagellum during sperm transit through the epididymis in many vertebrate species [4]. This structure was first reported by Retzius in 1909 and was termed the cytoplasmic droplet (CD) [5]. Earlier studies have described that the positional change of CDs during epididymal transition from the neck region and gradually down to the junction of mid-principal piece coincides with spermatozoa maturation [3]. Biochemical analyses revealed that CDs possess activities of lysosomal and glycolytic enzymes in bovine, boar, ram and rat [6–9]. Ultrastructral studies of the CD revealed that it consists of flattened membranous and vesicular components [6,9], resembling the saccular elements of the endoplasmic reticulum and Golgi apparatus [3,10]. Recent studies point to a potential role of CDs in volume regulation by allowing water entrance/exit so that spermatozoa can adapt to osmolality changes and maintain membrane integrity during their transit through the epididymis and through the female reproductive tract [11–13]. However, the osmoadaptation function remains controversial because ejaculated spermatozoa generally do not possess CDs although the osmolality in the female reproductive tract is much lower than in the cauda epididymis or semen (~300mosmol/l in the uterine cavity *vs.* ~440 mosmol/l in the cauda epididymis) [13]. Nevertheless, the presence of CDs on ejaculated spermatozoa appears to be adversely correlated with sperm fertility in both animals and humans [14–18]. Ejaculated spermatozoa of domestic animals containing a high proportion with proximal CDs are correlated with reduced *in vitro* fertilization and artificial insemination outcome [17,19–22]. In humans, data are controversial because some studies have associated the presence of CDs on ejaculated spermatozoa with poor in vitro fertilization (IVF) and intracytoplasmic sperm injection (ICSI) outcome, whereas some insist that CDs represent a normal structure of normal spermatozoa in ejaculates [23]. Our recent data suggest that the CD is a normal organelle exclusively present on epididymal spermatozoa, and normal CD morphology and location correlate with motility potential during epididymal sperm maturation [24]. Abnormal CDs, e.g. a complete lack of CDs or ectopic CDs, is indicative of defective spermiogenesis [24]. 

To shed light on the true physiological role of the CD, we purified CDs from epididymal spermatozoa by discontinuous sucrose gradient centrifugation and performed proteomic analyses on proteins highly enriched in CDs using mass spectrometry. We found that the majority of CD-enriched proteins are enzymes involved in energy metabolism. Functional analyses suggest that CDs appear to play an important role in priming spermatozoa for motility and fertility competence by serving as an energy-producing and “fueling” device to support molecular and cellular events essential for epididymal sperm maturation. 

## Results

### Intact cytoplasmic droplets can be isolated from epididymal spermatozoa

**Figure 1:**
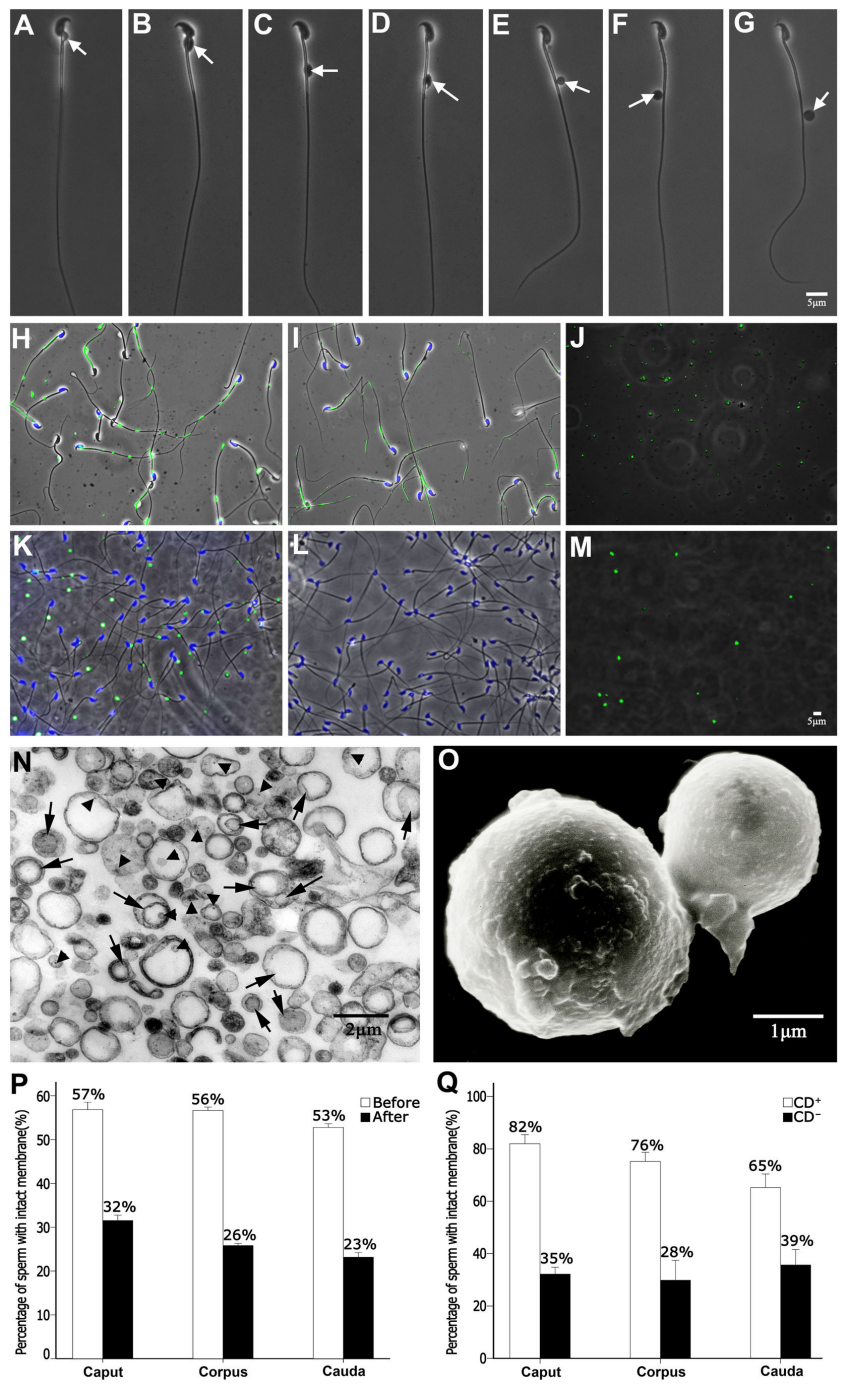
Purification of cytoplasmic droplets (CDs) using murine epididymal spermatozoa. (**A**-**G**) Phase contrast microscopic images showing epididymal spermatozoa with CDs (arrows) at various positions along sperm flagella. The same magnification was used for panels A-G (Scale bar, 5μm). (**H**-**J**) Epididymal spermatozoa with plasma membrane tagged with the membrane-bound form of EGFP (mG) from adult *Prm1-Cre; mTmG* ^*+/tg*^ male mice before (H) and after (I) sucrose gradient centrifugation-based CD removal, and the fluorescent microscopic images of purified CDs (J) are shown. The same magnification was used for panels H-J (Scale bar, 5μm). (**K**-**M**) Immunohistochemical analyses of SPEM1, a CD marker protein, on epididymal spermatozoa before (K) and after (L) sucrose gradient centrifugation-based CD removal, and the fluorescent microscopic images of purified CDs (M). The same magnification was used for panels K-M (Scale bar, 1μm). (**N**) A representative transmission electron microscopic image showing purified murine CDs, which are mostly spherical structures with continuous membrane enclosure containing dense granules (arrowheads) and membranous vesicles (arrows) (Scale bar, 2μm). (**O**) A representative scanning electron microscopic image of two purified murine CDs (Scale bar, 1μm). (**P**) Percentage of epididymal spermatozoa with intact plasma membrane before and after centrifugation-based CD purification. Data from triplicates (n=3) are presented as means ± SEM. (**Q**) Percentage of epididymal spermatozoa with (CD+) or without (CD-) CD displaying intact plasma membrane. Data from triplicates (n=3) are presented as means ± SEM.

CDs can be found in any position along the sperm flagella in the epididymis, but the most common location is the middle piece and mid-principal piece junction in mice (Figure 1A-G). We purified CDs from epididymal spermatozoa using a previously reported method [6], which was based on the fact that gentle physical shearing forces generated by the discontinuous sucrose gradient centrifugation can detach CDs from sperm flagella and create CD-enriched isodensity layers in the sucrose gradient. To verify the purity of CDs, we used spermatozoa from *Prm1-Cre; mTmG* ^*+/tg*^ male mice because the sperm plasma membrane was labeled with membrane-tagged eGFP (mG) in these mice (Figure 1H) [25–27]. After CD purification, the remaining spermatozoa were devoid of CDs (Figure 1I), and the CD fraction contained only mG-positive particles (Figure 1J). We also performed immunofluorescent staining to detect SPEM1, a marker protein for CDs [28–30]. CDs were detected in the majority of the caudal epididymal spermatozoa prior to the CD purification procedure (Figure 1K). After centrifugation, all spermatozoa lost their CDs (Figure 1L), and the purified CDs were mostly SPEM1-positive (Figure 1M). By counting particles with or without green signals in the CD fractions, we determined that the purity of isolated CDs was >95% (Figure 1J & 1M). 

 We also performed both transmission electron microscopy (TEM) and scanning electron microscopy (SEM) on purified CDs (Figure 1N & 1O). Both TEM and SEM showed that CDs were spherical and in variable sizes ranging from 0.5 to 2.0μm in diameter (Figure 1N and 1O). This is consistent with our observation that CDs on spermatozoa collected from different regions of the epididymis display variable sizes under phase contrast microscope (Figure 1A-1G). TEM also revealed that CDs possessed intact membrane enclosure, in which dense granules (arrowheads in Figure 1N) and membranous vesicles (arrows in Figure 1N) were clearly visible. Some SEM images also documented single indentations on one side of the CDs, presumably representing the re-sealed defects caused by CD detaching from the sperm flagella (Figure S1 in File S1). 

### Purified CDs have intact membranous enclosure, and so do the spermatozoa after CD removal

CDs were removed from the epididymal spermatozoa due to the shearing forces generated by discontinuous sucrose gradient centrifugation [6]. We initially assumed that spermatozoa after the CD removal would have membrane breakage. We examined the plasma membrane integrity using eosin-nigrosin staining [31]. While ~55% of total epididymal spermatozoa possessed intact plasma membrane (i.e. eosin Y-negative) (Figure 1P), ~30% of spermatozoa that had undergone centrifugation-based CD removal were also eosin Y-negative (Figure 1P), suggesting that membrane breakage occurred to only ~20-25% spermatozoa during the CD removal process. This implies that spermatozoa can repair the plasma membrane when CDs detached from the flagella, and this notion is further supported by the fact that in mice, almost all normal spermatozoa shed their CDs during ejaculation, and most (~60%) of the ejaculated spermatozoa remain membrane-intact and thus are motile. Epididymal spermatozoa without CDs account for ~30-45% of total epididymal spermatozoa [24]. Interestingly, we found that while ~70-80% of CD-bearing epididymal spermatozoa displayed intact plasma membrane, ~30% of CD-free epididymal spermatozoa were also membrane-intact (Figure 1Q). The proportion of CD-free epididymal spermatozoa with intact membrane was similar to that of spermatozoa after centrifugation-based CD removal (Figure 1P), further supporting the notion that spermatozoa are capable of repairing the membrane defects during CD detachment under both physiological (i.e. during spermiation) and experimental (i.e. sucrose gradient centrifugation-based CD isolation described herein) conditions. Hence, we used spermatozoa after centrifugation-based CD removal to represent those CD-free spermatozoa naturally present in the epididymis as negative controls in this study. 

### CDs are enriched with energy metabolic, especially glycolytic enzymes

**Figure 2:**
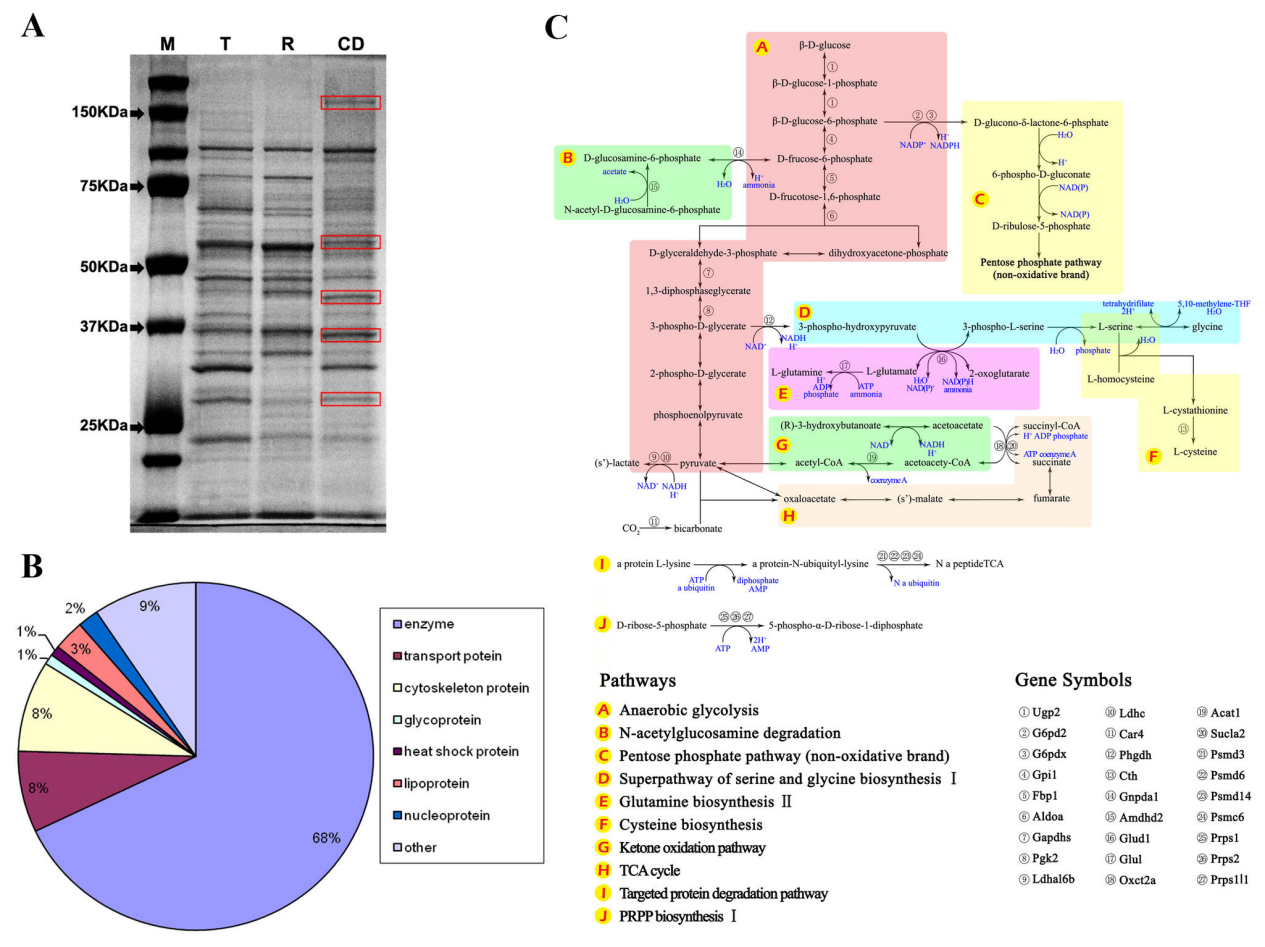
Proteomic analyses of proteins enriched in the cytoplasmic droplets (CDs) using mass spectrometry. (**A**) A representative SDS-PAGE gel showing the major protein bands (framed in red) that are either uniquely or predominantly detected in purified CDs. M, protein size marker; T, total epididymal spermatozoa; R, remaining spermatozoa after CD removal; CD, purified CDs. (**B**) A pie chart showing distribution of the 8 categories of proteins highly enriched in CDs and identified by mass spectrometry. (**C**) 27 out of 71 enzymes identified are those involved in metabolic pathways for energy production or phosphorylation.

The classic, substrate-based biochemical analyses performed >40 years ago have suggested that CDs display activities of lysosomal and glycolytic enzymes [6–9]. Despite the wide application of next-generation proteomic technologies, the complete proteome of CDs remain undefined. Given that CDs may contain many proteins that are also present in other parts of spermatozoa (e.g. outer dense fiber, plasma membrane, flagellum, etc.), we chose to identify proteins that are unique to or more abundant in CDs. We isolated proteins from total epididymal spermatozoa prior to CD purification (lane labeled with “T” for total spermatozoa in Figure 2A), after CD removal (lane with “R” for remaining CD-free spermatozoa in Figure 2A), and purified CDs. On SDS-PAGE gels, five bands representing the proteins unique or highly enriched in CDs were cut out and subjected to mass spectrometry (MS) for protein identification. MS analyses identified a total of 105 proteins with high confidence (>90% at p<0.05, Table S1 in File S1) and these proteins can be divided into 8 categories, including enzymes, cytoskeleton proteins, transport proteins, glycoproteins, heat shock proteins, lipoproteins, nucleoproteins and other miscellaneous proteins (Figure 2B). Consistent with previous reports suggesting that CDs are enzymatically active [6–9], 71 out of the 105 proteins identified (~68%) were either known enzymes or proteins with enzymatic activities, many of which have been reported to be involved in sperm functions (Table S2 in File S1). For example, lactate dehydrogenase isoform C (LDHC) and phosphoglycerate kinase 2 (PGK2) were both known for their involvement and necessity for sperm energy production through anaerobic glycolysis [32–34]. We further mapped 27 CD-enriched enzymes to 10 known metabolic pathways involved in energy production and phosphorylation (Figure 2C). Among these metabolic enzymes, 10 can catalyze anaerobic glycolysis, which is known to be the primary source of ATP production essential for sperm protein tyrosine phosphorylation, hyperactivated sperm motility and sperm capacitation in mammals [35–38]. 

### CDs are required for further distribution of glycolytic enzymes during epididymal sperm maturation

**Figure 3:**
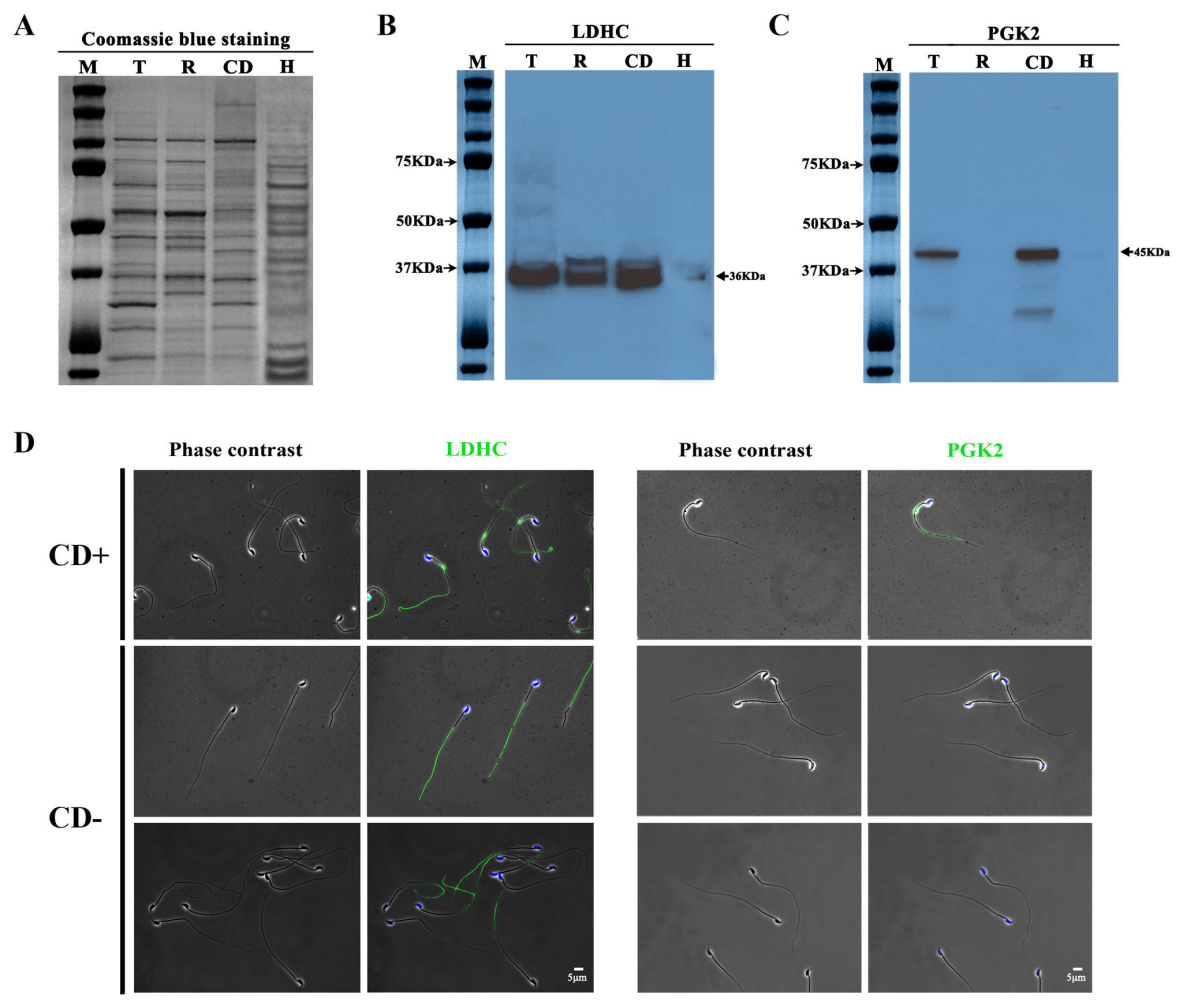
Enrichment of lactate dehydrogenase C (LDHC) and phosphoglycerate kinase (PGK2) in cytoplasmic droplets (CDs) and CD-dependent localization of LDHC and PGK2 in epididymal spermatozoa. (**A**) A representative SDS-PAGE gel showing equal loading of proteins (8 μg/lane) isolated from total epididymal spermatozoa (T), epididymal spermatozoa after CD removal (R), purified CDs (CD) and heart (H). M, protein size marker. (**B**) A representative Western blot result showing levels of LDHC in total epididymal spermatozoa (T), epididymal spermatozoa after CD removal (R), purified CDs (CD) and heart (H). (**C**) A representative Western blot result showing levels of PGK2 in total epididymal spermatozoa (T), epididymal spermatozoa after CD removal (R), purified CDs (CD) and heart (H). (**D**) Immunofluorescent staining of LDHC and PGK2 in CD-bearing (upper panels), CD-free (middle panels) epididymal spermatozoa form the caput, and epididymal spermatozoa after centrifugation-based CD removal (lower panels). All panels were in the same magnification (Scale bar, 5μm).

We further validated the enrichment of both LDHC and PGK2 in CDs by Western blot analyses (Figure 3). We loaded equal amount of proteins (8μg/lane) isolated from total epididymal spermatozoa (lanes labeled with “T” in Figure 3A-3C), epididymal spermatozoa after CD removal (lanes labeled with “R” in Figure 3A-3C), and purified CDs (lanes labeled with “CD” in Figure 3A-3C). Higher levels of LDHC were detected in CD-bearing spermatozoa and in purified CDs compared to those in spermatozoa after CD removal (Figure 3B). Unlike LDHC, PGK2 was much more abundant in CDs than in CD-bearing total spermatozoa, but barely detectable in spermatozoa with CDs removed (Figure 3C). To further examine the localization of LDHC and PGK2 in spermatozoa, cauda epididymal sperm smears were used for immunofluorescent staining using LDHC and PGK2 antibodies (Figure 3D). LDHC was detected in CDs and also along the entire length of flagella of CD-bearing cauda epididymal spermatozoa (upper left two panels in Figure 3D), whereas CD-free epididymal spermatozoa (middle left two panels in Figure 3D) and spermatozoa after CD removal (lower left two panels in Figure 3D) only displayed LDHC staining in the principal or end piece of the flagella. LDHC was absent in the neck and the middle piece of cauda epididymal spermatozoa. While PGK2 was detected in CDs and along the entire flagella of CD-bearing spermatozoa (upper right two panels in Figure 3D), PGK2 was hardly detectable in the CD-free epididymal spermatozoa (middle right two panels in Figure 3D) and spermatozoa after CD removal (lower right two panels in Figure 3D). These localization patterns are consistent with the Western blot data, demonstrating a lack of LDHC localization in the middle piece of spermatozoa, and a loss of PGK2 localization to the sperm flagella when CDs are absent. 

### CDs are required for mitochondrial activation and ATP production during epididymal sperm maturation

**Figure 4:**
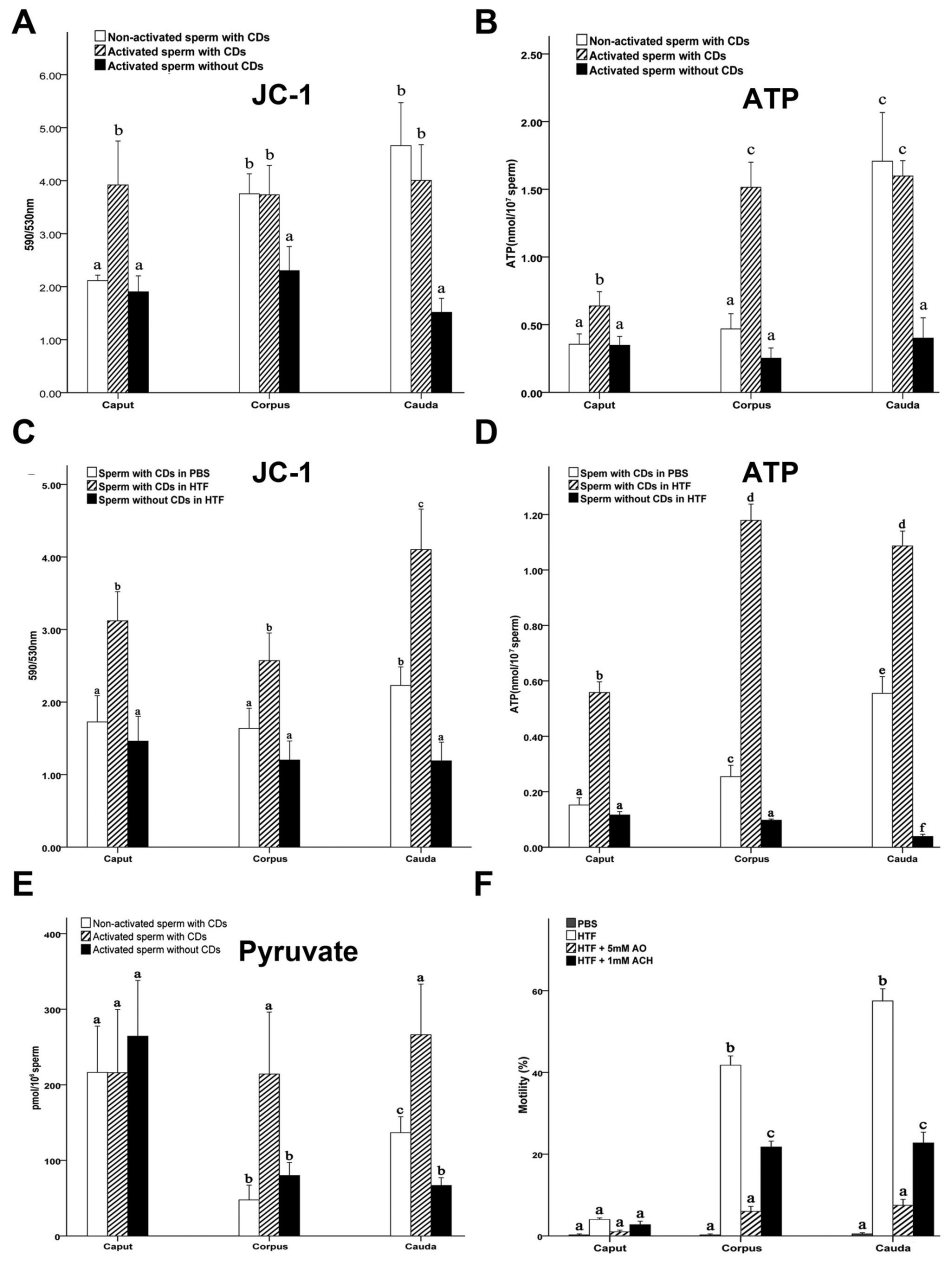
CDs appear to be required for normal mitochondrial activation and ATP production during epididymal sperm maturation. (**A**) Mitochondrial membrane potential in murine caput, corpus and cauda epididymal spermatozoa with or without CDs. Data are presented as mean ± SEM. Bars with different letters are significantly different (p<0.05, n=5). (**B**) ATP levels in murine caput, corpus and cauda epididymal spermatozoa with or without CDs. Data are presented as mean ± SEM. Bars with different letters are significantly different (p<0.05, n=5). (**C**) Mitochondrial membrane potential in monkey caput, corpus and cauda epididymal spermatozoa with or without cytoplasmic droplets (CDs). Data are presented as mean ± SEM. Bars with different letters are significantly different (p<0.05, n=5). (**D**) ATP levels in monkey caput, corpus and cauda epididymal spermatozoa with or without CDs. Data are presented as mean ± SEM. Bars with different letters are significantly different (p<0.05, n=5). (**E**) Pyruvate levels in murine caput, corpus and cauda epididymal spermatozoa with or without CDs. Data are presented as mean ± SEM. Bars with different letters are significantly different (p<0.05, n=5). (**F**) Effects of 5mM Ammonium Oxalate (AO) and 1mM α-Chlorohydrin (ACH) on the development of progressive motility of murine caput, corpus and cauda epididymal spermatozoa. Data are presented as mean ± SEM. Bars with different letters are significantly different (p<0.05, n=4).

It has been demonstrated that mitochondria of caput epididymal spermatozoa are largely inactive and gradually become activated when spermatozoa reach the corpus and cauda epididymidis in mice [39,40]. Indeed, we found that in mice, sperm mitochondrial activity, as reflected by the membrane potential, was much lower in the caput than in the corpus and cauda when incubated in non-activating medium (Figure 4A). When mouse epididymal spermatozoa (~60-70% with CDs) were activated with HTF, mitochondrial activity was more than doubled in all three regions (caput, corpus and cauda) of the epididymis (Figure 4A). However, the mitochondrial activity of epididymal spermatozoa without CDs remained at lower levels comparable to those of non-activated caput CD-bearing spermatozoa (Figure 4A). In non-activating medium (e.g. PBS), ATP levels were much lower in the caput and corpus spermatozoa, and a ~4-6-fold increase was observed in cauda epididymal spermatozoa, suggesting that ATP production is significantly enhanced when spermatozoa reach the cauda epididymis in mice (Figure 4B). Upon activation, ATP production was enhanced by ~2-4 folds in spermatozoa from all three regions of the epididymis with much higher levels in the corpus and cauda spermatozoa than in the caput spermatozoa in mice (Figure 4B). 

We further analyzed mitochondrial activity and ATP production in monkey epididymal spermatozoa (Figure 4C,D). Similar to murine epididymal spermatozoa, monkey epididymal spermatozoa displayed significant increases (~1.8-fold) in mitochondrial activity once activated in the HTF-HEPES medium, whereas non-activated spermatozoa demonstrated much lower mitochondrial activities (Figure 4C). Epididymal spermatozoa without CDs displayed baseline mitochondrial activity even after incubation in the HTF-HEPES medium, suggesting that CDs are also required for sperm mitochondrial activation in monkeys. ATP levels were ~2-4-fold higher in activated than in non-activated monkey epididymal spermatozoa although sperm ATP levels generally increased from the caput to cauda (Figure 4D). However, epididymal spermatozoa without CDs displayed minimal ATP levels (Figure 4D). Together, failure of CD-free epididymal spermatozoa to undergo mitochondrial activation and ATP production may explain why spermatozoa without CDs are generally immotile despite the fact that a significant proportion of them still possess intact plasma membrane (Figure 1P,Q, Videos S1 and S2 in File S1). 

 We further examined pyruvate levels in epididymal spermatozoa (Figure 4E) because pyruvate represents an intermediate substrate of both glycolysis and the tricarboxylic acid cycle (TCA, also called citric acid cycle, or Krebs’ cycle) [36,41,42]. Pyruvate can be converted into either lactate in anaerobic glycolysis, or acetyl-coenzyme A in the TCA cycle for ATP production. Pyruvate levels were relatively higher in caput spermatozoa than in corpus and cauda spermatozoa regardless of the presence/absence of CDs (Figure 4E). However, pyruvate levels decreased in non-activated spermatozoa from the corpus and cauda compared to those from the caput (Figure 4E). This may suggest that pyruvate is being actively utilized during sperm epididymal transition from the caput to the corpus and then to the cauda. Once activated in the pyruvate-free TYH medium, CD-bearing epididymal spermatozoa maintained higher levels of pyruvate, whereas pyruvate levels dropped in CD-free spermatozoa of the corpus and the cauda epididymis (Figure 4E), suggesting that a sustained supply of glycolytic substrates may require CDs. 

### Glycolytic inhibitors can suppress the development of progressive motility of epididymal spermatozoa

Given that spermatozoa without CDs cannot develop motility and CDs are enriched with glycolytic enzymes, it is highly likely that CDs act as temporary energy-producing devices at least in part through glycolysis. If so, one would expect that glycolytic inhibitors would hamper the development of progressive motility of epididymal spermatozoa. We, therefore, added two glycolytic inhibitors, ammonium oxalate (AO) and α-Chlorohydrin (ACH), into HTF-HEPES medium during epididymal sperm activation (Figure 4F). Caput epididymal spermatozoa developed little or no progressive motility, which is normal because these caput epididymal spermatozoa have not undergone the maturation process and thus, are unable to develop progressive motility [43]. In contrast, after 60-min incubation in HTF-HEPES medium at 37°C, ~42% and ~56% of corpus and cauda spermatozoa displayed progressive motility, respectively (Figure 4F). In corpus and cauda epididymal spermatozoa incubated in HTF-HEPES containing 5mM AO, only ~6% and 8% developed progressive motility after 60-min activation, respectively (Figure 4F). When incubated with HTF-HEPES containing 1mM ACH, ~21% and 23% of corpus and cauda spermatozoa displayed progressive motility, respectively (Figure 4F). Since these epididymal spermatozoa mostly (>60%) possess CDs with abundant glycolytic enzymes, the suppressive effects on the development of sperm progressive motility at least in part result from inhibition of enzymatic activity in both CDs and the flagellum by the glycolytic inhibitors. These data further support the idea that CDs may represent a temporary energy-producing and fueling device essential for making spermatozoa competent for developing progressive motility upon ejaculation. This notion is also consistent with our observation that epididymal spermatozoa with CDs developed vigorous progressive motility once collected in HTF medium, whereas spermatozoa without CDs or with their CDs physically removed (e.g. by gradient sucrose centrifugation as in this report) were completely immotile (Videos S1 and S2 in File S1). 

## Discussion

The CD appears to be an evolutionarily conserved organelle unique to epididymal spermatozoa as CDs have been observed in >30 vertebrate species from fish to primates and humans (Table S3 in File S1). The outer membrane of the CD is continuous with the plasma membrane of the sperm flagellum, and this feature has been used for anchoring patch-clamps for measuring the membrane potential of spermatozoa [44]. The finding that the purified CDs possess continuous and intact membrane enclosure suggests that CDs can reseal the defects at the detaching points. While the detached CDs can repair their membrane, spermatozoa also appear to be able to fix their membranous defects at the CD-detaching sites, leading to intact plasma membrane in most of the ejaculated spermatozoa after CD removal during epididymal maturation or ejaculation. Interestingly, ~30% of the CD-free epididymal spermatozoa possess intact membrane and a similar proportion of spermatozoa after CD removal also have intact membrane. These data suggest that the repair can occur both *in vivo* and *in vitro*, and thus, the membrane repair mechanism appears to lie within spermatozoa and CDs. It would be interesting to further explore how spermatozoa fix the membranous breakage when CDs are shed.

The fact that CDs are present exclusively on epididymal spermatozoa suggests that CDs may have a role unique to epididymal sperm maturation. Earlier studies identified activities of several classes of enzymes, including lysosomal hydrolases (e.g. acid/alkaline phosphatase, beta-glucuronidase, nucleotide phosphatase) and intermediate metabolic enzymes (e.g. transaldolase, lactic dehydrogenase and glutamate oxaloacetate transaminesase) in CDs of rat, rabbit, ram and bovine spermatozoa [6–10,45–47]. Most of those enzymes are confirmed in our proteomic analyses, thus supporting the earlier hypothesis that CDs may have a role in energy metabolism during epididymal sperm maturation [6-10]. The CD purification procedure provided us with an opportunity to evaluate differences between CD-bearing and CD-free epididymal spermatozoa. The notion that CDs are required for ATP production is consistent with and supported by our findings that CDs are enriched with various metabolic enzymes involved in ATP production. The dynamic changes of pyruvate levels in CD-bearing spermatozoa further support that CDs may represent an organelle with active energy metabolism, which can be largely abolished when CDs are absent or removed. Taken together, CDs appear to serve as an energy source, which provides energy (i.e. ATPs) required for the ongoing maturation of epididymal spermatozoa. 

Numerous molecular and cellular events occur during epididymal sperm maturation, including exposure of surface lipids and proteins (e.g. receptors and ion channels) followed by modifications (e.g. phosphorylation and glycosylation), changes in the acrosome membrane, modification of the nucleus, incorporation of glycolytic enzymes into the flagellum, etc. [14,40]. Typical protein modifications during epididymal sperm maturation include tyrosine phosphorylation and serine/threonine phosphorylation [48–51]. Interestingly, many phosphatase are also enriched in CDs, such as PPP1CC, PRSS21 and PRSS52, which are all involved in protein serine/threonine phosphorylation, and SORD, a protease associated with protein tyrosine phosphorylation [52–54], was also highly enriched in CDs. Thus, CDs also appear to provide modifying enzymes for sperm protein modifications. The finding that CDs are required for the “fueling” process through which materials required for energy metabolic pathways (e.g. enzymes and substrates) are integrated into and stored in various sub-compartments of the flagellum is consistent with the fact that both glycolytic inhibitors and genetic inactivation of key glycolytic enzymes (e.g. PGK2 and GAPDS) can abolish sperm competence for developing progressive motility [34,35]. 

It has been suggested that sperm mitochondria remain largely inactive during epididymal sperm maturation because minimal membrane potential can be detected [39,40], and our data confirmed this phenomenon. Interestingly, mitochondria become gradually activated during epididymal sperm maturation, and by the time spermatozoa reach the cauda epididymis, the mitochondrial membrane potential reaches to levels comparable to that of HTF-activated spermatozoa. Physiologically, this may suggest that mitochondria undergo a “priming” process, through which inactive mitochondria become activated and thus competent for producing energy through the oxidative phosphorylation system in conjunction with active glycolysis within the flagellum. This is also supported by a previous report showing that the knockout male mice lacking transaldolase (TAL), one of many enzymes enriched in CDs, displayed abnormal mitochondria with no detectable membrane potential, and consequently immotile spermatozoa and male infertility [55]. Given that mitochondria of epididymal spermatozoa are mostly inactive, it is physiologically plausible that CDs function as a temporary energy source for most, if not all, of the energy (e.g. ATP) producing pathways, which are required for all cellular and molecular events during epididymal sperm maturation. Therefore, CDs appear to be a transient organelle essential for epididymal sperm maturation by acting as an energy-producing device. In that sense, CDs function to “prime” the epididymal spermatozoa for motility and fertilization competence. Since this study only analyzed proteins highly enriched in CDs, the full proteome needs to be defined in the near future to further support this and other potential functions of CDs. 

In summary, our data suggest that CDs represent a temporary device essential exclusively for sperm epididymal maturation by providing the energy required. Once ejaculated, spermatozoa lose their CDs through an unknown mechanism, which probably involves mechanical shearing or enzymatic activities. Nevertheless, ejaculated spermatozoa can be fully functional without CDs because they have become fully competent in generating sustainable energy to support the long-lasting motility using enzymes and substrates that have been integrated into the flagellum and activated mitochondria, all of which require CDs during sperm epididymal maturation. Because CDs are essential for epididymal sperm maturation, methods that can eliminate CDs or inhibit CD functions should represent an effective means of male contraception. 

## Materials and Methods

### Laboratory animals

Mice were housed in a temperature- and humidity-controlled animal facility in the University of Nevada, Reno, with free access to water and food. Adult male mice of 8-12 weeks of age were used for collecting epididymal spermatozoa. The animal protocol was approved by the Institutional Animal Care and Use Committee (IACUC) of the University of Nevada, Reno.

Cynomolgus monkey (*Macaca fascicularis*) epididymidis were obtained from control animals used in clinical trials in Charles River Laboratories, Preclinical Services (Sparks, NV), and no monkeys were killed specifically for experiments described in this paper. Cynomolgus monkeys were housed indoor in individual primate cages containing fabric hammocks, wooden perches and nest boxes, and fed twice a day with a standard commercial primate chow with water available ad libitum. Lights were on from 6:30 to 18:30 h, and room temperature and humidity were maintained at ~24°C and 30–70%, respectively. The protocol for euthanizing monkeys was approved by the Institutional Animal Care and Use Committee (IACUC) of Charles River Laboratories, and assures compliance with the United States Department of Agriculture (USDA), Public Health Service (PHS) Office of Laboratory Animal Welfare (OLAW) Policy and the Animal Welfare Act. Adult male monkeys were sedated with Ketamine (10 mg/kg) *via* intramuscular injection to the quadriceps. They were then given Beuthanasia-D^®^ solution containing pentobarbital sodium and phenytoin sodium (200mg/kg B.W.) *via* intravenous injection followed by exsanguination. The epididymidis were dissected, placed in cold KRBS and transported to our lab within 30 minutes after dissection.

### Isolation of cytoplasmic droplets

Adult C57Bl/6J mice were euthanized and the epididymidis were dissected and transferred into 2.5ml of a dissection medium (0.15M KCl solution containing 0.01M Tris-HCl, pH7.1). The epididymidis were further dissected into smaller pieces (5mm X 5mm) followed by incubation in a humidity incubator at 37°C for ~10min. The spermatozoa-containing supernatants (~2 ml) were collected and subjected to cytoplasmic droplet purification using a discontinuous sucrose density gradient centrifugation method [6] with some modifications. Briefly, ~2ml of sperm suspension were layered over a discontinuous sucrose gradient composed of 2ml of 0.25M sucrose on top of 3ml of 1M sucrose. After centrifugation for 20min at 1,500g, the top 1ml of the 0.25M sucrose layer was removed, and the remainder of the 0.25M sucrose layer and materials located at the interface with the 1M sucrose layer were carefully transferred to a new 1.5ml centrifuge tube followed by centrifugation at 1,200g for 10 min. The CD-enriched supernatant was transferred to a new 1.5ml centrifuge tube followed by centrifugation at 12,500g for 20min at 4°C to yield the CD pellet. The pellet at the bottom of the 1M sucrose layer contained spermatozoa without CDs and was collected into a new 1.5ml centrifuge tube for analyses. 

### Electron microscopy

The CD pellet was fixed in ice-cold 2.5% (v/v) glutaraldehyde in 0.12M cacodylate buffer, pH 7.4 overnight at 4°C. For transmission electron microscopy (TEM), samples were then washed with 1X PBS three times and post-fixed with ice-cold 1.5% osmium tetroxide in 0.12M cacodylate buffer, pH 7.4, for 1 h at 4°C. Dehydration was performed by first incubating the pellet in 50% and 70% ethanol sequentially, followed by 80%, 90% and 100% acetone. After dehydration, samples were embedded in Epon812, and thin sections (50-70nm) were cut and stained with uranyl acetate and lead citrate. The ultrastructure of the samples was observed and photographed using a transmission electron microscope (JEM-1200EX, Tokyo, Japan) at 80kV. For preparing samples for scanning electron microscopy (SEM), the fixed CDs pellets were first washed with 1XPBS for 3 times and 5min each, followed by dehydration in 50%, 90% and 100% ethanol, sequentially. Then CD pellets were resuspended in 100% isoamyl acetate, dropped onto polylysine-coated glass coverslip, and dried in a critical point drier (JEOL, Tokyo, Japan) in liquid CO_2_. Selected regions of the glass were cut and mounted on to stubs for observation under a scanning electron microscope (JSM-T300, Tokyo, Japan) at 20kV of filament intensity.

### Immunofluorescence microscopy

Immunofluorescent staining was performed as described [56]. Briefly, cauda epididymal spermatozoa and the purified CD suspensions were spread onto Superfrost Plus slides (Fisher Scientific, Hampton, NH) and air-dried. Smear slides were fixed in 4% paraformaldehyde, permeabilized with 0.5% Triton X-100 in phosphate-buffered saline (PBS) for 2min at 4°C, washed with 1×PBS for 3 times (5min/wash), and incubated with 100µl Blocking Buffer (30µl FBS + 30 µl normal goat serum + 940µl 1%BSA in PBS) at RT for 1h. Primary antibody incubation was performed in a humidified chamber at 4°C overnight. The rabbit anti-LDHC (a generous gift from Dr. Erv Goldberg, Ph.D., Northwestern University) and the rabbit anti-PGK2 (from Dr. Deborah O’Brien, Ph.D., University of North Carolina) polyclonal antibodies were used at dilutions of 1:4,000 and 1:2,000, respectively. The rabbit anti-SPEM1 polyclonal antibody [30] was used at a dilution of 1:1,000. After overnight primary antibody incubation, slides were moved to room temperature for ~30min before 3 washes with 1×PBS (10 min/ wash). Slides were then incubated with the fluorescence-conjugated, species-specific secondary antibodies (Molecular Probes) at room temperature for 1h with gentle shaking, followed by 3 1×PBS washes (10 min/ wash). Finally, slides were counterstained with 4′, 6-diamidino-2-phenylindole (DAPI, Sigma) to visualize nuclei. Fluorescence microscopic imaging was conducted using a fluorescence microscope (Carl Zeiss, HAL100).

### Eosin-nigrosin staining

Sperm membrane integrity was assessed by eosin-nigrosin staining using the Sperm Viability Stain Kit (Fertility Solutions, Cleveland, OH, Cat^#^SA200) according to the manufacturer's protocol. 

### Identification of proteins enriched in cytoplasmic droplets

The total epididymal spermatozoa, the isolated cytoplasmic droplets, and the spermatozoa after CD removal by gradient sucrose centrifugation, were solubilized in lysis buffer (25mM Tris-HCl, pH 7.6, 150mM NaCl, 1%(v/v) NP-40, 0.1% SDS, and protease inhibitors). Protein concentrations were measured using a BCA kit (Pierce, Rockford, IL) with bovine serum albumin as the standard. Polyacrylamide electrophoresis (PAGE) was performed using 8µg proteins and 10% Bis-Tris large format gel (Criterion XT precast gel, Bio-Rad) with XT MOPS buffer (pH 6.9) (Bio-Rad, Hercules, CA) at 200V for 60min at 4°C. The molecular size was determined based upon dual color protein weight standards (Bio-Rad, Hercules, CA). The gel was stained with Coomassie brilliant blue solution (Colloidal Blue Staining Kit, Invitrogen, CA) for 4hr and de-stained in de-ionized water for at least 7hr. The bands on the gel unique to CDs were cut using an EXQuest spot cutter (Bio-Rad, Hercules, CA) and digested using Investigator ProPrep (Genomic Solutions, Ann Arbor, MI), according to a previously described protocol [57] with some modifications. Samples are washed twice with 25mM ammonium bicarbonate and 100% acetonitrile (ACN), reduced and alkylated using 10mM DTT and 100mM iodoacetamide followed by incubation with Trypsin (75ng/μl) in 25mM ammonium bicarbonate for 6hr at 37°C. Samples are prepared and spotted onto a MALDI (Matrix Assisted laser Desorption Ionization) target with ZipTipu-C18 (Millipore, Billerica; MA). Next, samples are aspirated and dispensed 3 times and eluted with 70% ACN, 0.2% formic acid and overlaid with 0.5μl 5mg/mL MALDI matrix (α-Cyano-4-hydroxycinnamic acid) and 10mM ammonium phosphate. All mass spectrometric data were collected using an ABI 4700 MALDI TOF/TOF (Applied Bio-systems, Foster City; CA).

### JC-1 assay

Sperm mitochondrial membrane potential was measured using a JC-1 assay kit (Molecular probes, Invitrogen, CA). Spermatozoa collected from the caput, corpus and cauda epididymidis were incubated at 37°C for 40min in either PBS (non-activated spermatozoa with CDs) or HTF-HEPES (activated spermatozoa with CDs). Epididymal spermatozoa after CD removal using sucrose gradient centrifugation were incubated at 37°C for 40min in HTF-HEPES medium (activated group without CDs). Fluorescence (excitation at 490nm and emission at 530 or 590 nm) detection was carried out in a microplate reader (SpectraMax Gemini EM, Molecular Device, Sunnyvale, CA). Ratios between red fluorescence (high membrane potential; excitation 490 nm, emission 590 nm) and green fluorescence (low membrane potential; excitation 490 nm, emission 530 nm) were calculated.

### Measurement of ATP levels

Sperm ATP levels were measured as described [33] with slight modifications. Spermatozoa collected from the caput, corpus and cauda epididymidis were incubated at 37°C for 40min in either PBS (non-activated spermatozoa with CDs) or HTF-HEPES (activated spermatozoa with CDs). Epididymal spermatozoa after CD removal using sucrose gradient centrifugation were incubated at 37°C in for 40min in HTF-HEPES medium (activated spermatozoa without CDs). Spermatozoa were centrifuged at 1,000g for 5 min. The pellet was re-suspended in a buffer (100mM Tris-HCl, 4mM EDTA, pH7.8) and boiled for 5 min. The sample was then centrifuged at 10,000g for 5 min, and aliquots of the supernatant were used for measuring ATP levels using an ATP Bioluminescence Assay kit CLS II according to the manufacturer’s instructions (CLSII, Roche Applied Science, Indianapolis, IN).

### Measurement of pyruvate levels

Pyruvate levels were determined using a commercial kit (BioVision, Mountain View, CA), which is based on an enzymatic reaction catalyzed by pyruvate oxidase and interactions of the reaction products with a probe to produce fluorescence. Spermatozoa collected from the caput, corpus and cauda epididymidis were incubated at 37°C for 4hr in either PBS (non-activated spermatozoa with CDs) or pyruvate-free TYH medium (activated spermatozoa with CDs). Epididymal spermatozoa after CD removal using sucrose gradient centrifugation were incubated at 37°C for 4hr in TYH medium (activated group without CDs). After incubation, spermatozoa were centrifuged and the pellet was resuspended in the assay buffer followed by fluorescence measurement (excitation at 535nm and emission at 590nm) using a microplate reader (SpectraMax Gemini EM). The pyruvate concentration of each sample was calculated based on a standard curve of pyruvate supplied by the kit.

### Western blot assays

Western blot analyses were performed as described [58]. Briefly, protein was extracted from total spermatozoa, spermatozoa after CD removal and purified CDs. Protein concentrations were measured using a BCA protein assay kit (Pierce Biotechnology, Indianapolis, IN). Protein samples (8µg/lane) were fractionated on 10% Tris–HCl polyacrylamide gels through electrophoresis and transferred onto nitrocellulose membranes (Bio-Rad). The membrane was then blocked with 5% non-fat milk. Primary antibody incubation was conducted at 4°C overnight. After three washes with TBST (1×TBS containing 0.1% Tween-20), membranes were incubated with a secondary antibody conjugated with horseradish peroxidase (1:1000, Amersham) for 1hr, followed by washing. An enhanced chemiluminescence detection kit (Amersham) was used for visualizing protein bands.

### Sperm motility and motile sperm counting

Progressive motility and total motility (flagellation *in situ* and progressive motility) were analyzed using the computer-assisted sperm analysis (CASA) system (version 14.0, Hamilton-Thorne Bioscience, Beverly, MA, USA). The parameters were set as follows: 60 frames/s (Hz), 30 frames per field. In this study, total motility (TMOT, %), progressive motility (PMOT, %), average path velocity (VAP, μm/s), straightness (STR, as VSL/VAP, %) were measured. Based on their VAP, spermatozoa were classified as rapid (VAP > 50 μm/s), medium (50 μm/s > VAP > 6 μm/s), slow (VAP < 6 μm/s), or static (VAP = 0). Spermatozoa with progressive motility displayed VAP ≥50 μm/s and STR ≥80%. Dual-sided analysis chamber slides (depth=20μm, Hamilton Thorne Research) were used for analyses. Spermatozoa from different regions of the epididymis (caput, corpus and cauda) were collected into PBS or HTF containing 1mM α-Chlorohydrin (MP Biomedicals, Cat#202178) or 5mM ammonium oxalate (Sigma-Aldrich, Cat#09898) followed by incubation at 37°C for 1hr. The sperm suspension was diluted to a concentration of 10,000 cells/μl and 5μl were loaded to a chamber slide for motility assays using CASA. 

### Statistical analyses

Data are presented as mean ± SEM, and statistical differences between datasets were assessed by one-way ANOVA using the SPSS16.0 software. Differences were considered significant at the level of *p* < 0.05. 

## Supporting Information

File S1 **Supplemental information is provided which contains one Figure (Figure S1), three Tables (Tables S1-S3) and two Videos (Videos S1 and S2).**
(PDF)
