# Supplementary material for: Proteomic Analyses Reveal a Role of Cytoplasmic Droplets as an Energy Source during Epididymal Sperm Maturation
Source: PLoS One. 2013 Oct 14;8(10):e77466. doi: 10.1371/journal.pone.0077466 (PMC3796460; doi:10.1371/journal.pone.0077466)
Supplement: File S1 — Supplemental information is provided which contains one Figure (Figure S1), three Tables (Tables S1-S3) and two Videos (Videos S1 and S2). (PDF) [file pone.0077466.s001.pdf]

Proteomic analyses reveal a role of cytoplasmic droplets as an energy source during sperm epididymal maturation

Shuiqiao Yuan[a,b], Huili Zheng[a], Zhihong Zheng[b], Wei Yan[a,1]

[a]Department of Physiology and Cell Biology, University of Nevada School of Medicine, Reno, NV, 89557; and [b]Department of Laboratory Animal Medicine, China Medical University, Shenyang, 110001, China

[1] Corresponding author. Email: wyan@unr.edu

Supplemental Information contains one Figure (Figure S1), three Tables (Tables S1-S3) and two Videos (Videos S1 and S2) files.

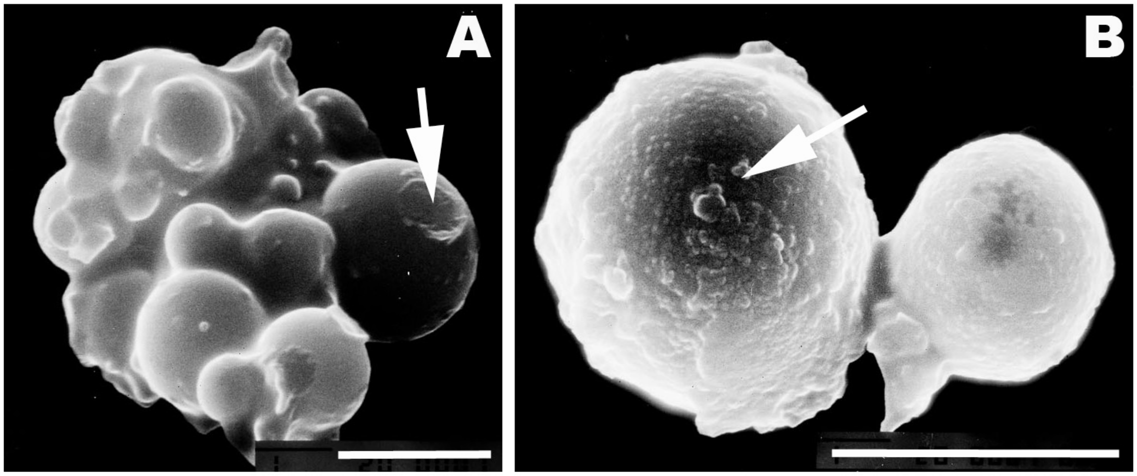

Figure S1. Scanning electron microscopic images of purified murine cytoplasmic droplets. Arrows point to indentations resembling the resealed defects at the detaching points when CDs come off the sperm flagella. Scale bar = 1μm

Table S1 Mass spectrometry-based identifiaction of proteins highly enriched in murine cytoplasmic droplets.

#	MS/MS View:Identified Proteins (105)	Accession Number	Molecular Weight	Protein Grouping Ambiguity	Dot_1_1	Dot_2_1	Dot_3_1	Dot_4	Dot_5_1	Dot_1_2	Dot_2_2	Dot_3_2	Dot_4_2	Dot_5_2
1	IPI:IPI00467457.3 Tax_Id=10090 Gene_Symbol=Ldhc L-lactate dehydrogenase C chain	IPI00467457	36 kDa	TRUE	91%	100%	100%	100%	100%		100%	100%	100%	100%
2	IPI:IPI00473320.2 Tax_Id=10090 Gene_Symbol=Actb Putative uncharacterized protein	IPI00473320	42 kDa	TRUE	75%	100%	100%	100%	100%	89%	76%	100%	100%	100%
3	IPI:IPI00224181.7 Tax_Id=10090 Gene_Symbol=Akr1b7 Aldose reductase-related protein 1	IPI00224181	36 kDa	TRUE				100%	100%		76%		100%	100%
4	IPI:IPI00228633.7 Tax_Id=10090 Gene_Symbol=Gpi1 Glucose-6-phosphate isomerase	IPI00228633	63 kDa			100%					100%	90%		
5	IPI:IPI00134191.3 Tax_Id=10090 Gene_Symbol=Slc2a3 Solute carrier family 2, facilitated glucose transporter member 3	IPI00134191	53 kDa		100%	100%	100%	100%	100%	100%	100%	100%	100%	100%
6	IPI:IPI00272690.2 Tax_Id=10090 Gene_Symbol=Ace Isoform Somatic of Angiotensin-converting enzyme	IPI00272690	151 kDa		100%	100%		89%		100%	100%		89%	
7	IPI:IPI00929768.1 Tax_Id=10090 Gene_Symbol=Pgk2 phosphoglycerate kinase 2	IPI00929768	45 kDa		91%	100%	100%	100%	100%		100%	100%	100%	100%
8	IPI:IPI00320217.9 Tax_Id=10090 Gene_Symbol=Cct2 T-complex protein 1 subunit beta	IPI00320217	57 kDa			100%	100%	89%			100%	90%	48%	
9	IPI:IPI00387281.1 Tax_Id=10090 Gene_Symbol=Oxct2a Succinyl-CoA:3-ketoacid-coenzyme A transferase 2A, mitochondrial	IPI00387281	56 kDa	TRUE		100%	78%				100%			
10	IPI:IPI00114593.1 Tax_Id=10090 Gene_Symbol=Actc1 Actin, alpha cardiac muscle 1	IPI00114593 (+1)	42 kDa	TRUE		100%	100%	89%	90%			100%	89%	92%
11	IPI:IPI00138399.9 Tax_Id=10090 Gene_Symbol=Heatr7b2 hypothetical protein LOC223825	IPI00138399	188 kDa		100%					100%				
12	IPI:IPI00460063.2 Tax_Id=10090 Gene_Symbol=Pcyox1 Prenylcysteine oxidase	IPI00460063	56 kDa			100%					100%			
13	IPI:IPI00387289.3 Tax_Id=10090 Gene_Symbol=Ces3 Carboxylesterase 3	IPI00387289	62 kDa			100%					100%			
14	IPI:IPI00331394.3 Tax_Id=10090 Gene_Symbol=Dnpep aspartyl aminopeptidase isoform a	IPI00331394	52 kDa			100%	100%				100%			
15	IPI:IPI00125705.1 Tax_Id=10090 Gene_Symbol=Tex101 Testis-expressed protein 101	IPI00125705	27 kDa					100%	100%				100%	100%
16	IPI:IPI00319965.3 Tax_Id=10090 Gene_Symbol=Psmd6 26S proteasome non-ATPase regulatory subunit 6	IPI00319965	46 kDa				100%					100%		
17	IPI:IPI00122815.3 Tax_Id=10090 Gene_Symbol=P4hb Putative uncharacterized protein	IPI00122815 (+1)	57 kDa			100%	100%				100%	100%		
18	IPI:IPI00125971.1 Tax_Id=10090 Gene_Symbol=Psmc6 26S protease regulatory subunit S10B	IPI00125971	44 kDa				100%					100%		
19	IPI:IPI00230108.6 Tax_Id=10090 Gene_Symbol=Pdia3 Protein disulfide-isomerase A3	IPI00230108	57 kDa			100%					100%			
20	IPI:IPI00554868.2 Tax_Id=10090 Gene_Symbol=Prps1 Ribose-phosphate pyrophosphokinase 1	IPI00554868 (+4)	35 kDa	TRUE				100%					100%	
21	IPI:IPI00228630.5 Tax_Id=10090 Gene_Symbol=Fbp1 Fructose-1,6-bisphosphatase 1	IPI00228630	37 kDa					100%					100%	
22	IPI:IPI00762897.2 Tax_Id=10090 Gene_Symbol=Uggt1 UDP-glucose:glycoprotein glucosyltransferase 1	IPI00762897	176 kDa		100%					100%				
23	IPI:IPI00314439.3 Tax_Id=10090 Gene_Symbol=Psmd3 26S proteasome non-ATPase regulatory subunit 3	IPI00314439 (+1)	61 kDa			100%					100%			
24	IPI:IPI00119495.3 Tax_Id=10090 Gene_Symbol=Prss21 Testisin	IPI00119495	36 kDa				100%	89%	100%			100%	89%	92%
25	IPI:IPI00273646.9 Tax_Id=10090 Gene_Symbol=Gm3272;LOC100042025;Gm12033;Gapdh;Gm10293;Gm10359;Gm2574;Gm5138 Glyceraldehyde-3-phosphate dehydrogenase	IPI00273646	36 kDa	TRUE		100%		100%			100%		100%	
26	IPI:IPI00225945.1 Tax_Id=10090 Gene_Symbol=Ldhal6b L-lactate dehydrogenase	IPI00225945	42 kDa	TRUE		83%	100%	100%				90%	100%	
27	IPI:IPI00626662.3 Tax_Id=10090 Gene_Symbol=Aldh1a1 Retinal dehydrogenase 1	IPI00626662	54 kDa			100%	100%	100%			100%	100%	100%	87%
28	IPI:IPI00124221.1 Tax_Id=10090 Gene_Symbol=Atp1b3 Sodium/potassium-transporting ATPase subunit beta-3	IPI00124221	32 kDa			100%	100%	100%	90%		100%	100%	100%	100%
29	IPI:IPI00115528.3 Tax_Id=10090 Gene_Symbol=Pls3 Plastin-3	IPI00115528 (+1)	71 kDa	TRUE		100%					100%			
30	IPI:IPI00119113.3 Tax_Id=10090 Gene_Symbol=Atp6v1b2 V-type proton ATPase subunit B, brain isoform	IPI00119113	57 kDa	TRUE		100%					100%			
31	IPI:IPI00225961.5 Tax_Id=10090 Gene_Symbol=Phgdh D-3-phosphoglycerate dehydrogenase	IPI00225961	57 kDa			100%					100%			
32	IPI:IPI00468124.1 Tax_Id=10090 Gene_Symbol=Ppp3cc Serine/threonine-protein phosphatase 2B catalytic subunit gamma isoform	IPI00468124	59 kDa	TRUE		100%					100%			
33	IPI:IPI00116277.3 Tax_Id=10090 Gene_Symbol=Cct4 T-complex protein 1 subunit delta	IPI00116277	58 kDa			100%	100%			91%	100%	100%		
34	IPI:IPI00228867.5 Tax_Id=10090 Gene_Symbol=G6pd2 Glucose-6-phosphate 1-dehydrogenase 2	IPI00228867	59 kDa	TRUE		100%					100%			
35	IPI:IPI00225668.1 Tax_Id=10090 Gene_Symbol=Fam78a Protein FAM78A	IPI00225668	32 kDa						100%					100%
36	IPI:IPI00114256.2 Tax_Id=10090 Gene_Symbol=Sypl Isoform 1 of Synaptophysin-like protein 1	IPI00114256 (+1)	29 kDa			83%			100%		100%			100%
37	IPI:IPI00118037.1 Tax_Id=10090 Gene_Symbol=Dpep3 Dipeptidase 3	IPI00118037	54 kDa			100%	100%	100%	100%		100%	100%	100%	100%
38	IPI:IPI00113262.1 Tax_Id=10090 Gene_Symbol=Psmd14 26S proteasome non-ATPase regulatory subunit 14	IPI00113262	35 kDa					100%					100%	
39	IPI:IPI00221540.1 Tax_Id=10090 Gene_Symbol=Erlin2 Erlin-2	IPI00221540	38 kDa				100%					100%		
40	IPI:IPI00469307.2 Tax_Id=10090 Gene_Symbol=Lrpap1 Alpha-2-macroglobulin receptor-associated protein	IPI00469307	42 kDa				100%					100%		
41	IPI:IPI00261627.1 Tax_Id=10090 Gene_Symbol=Sucla2 Succinyl-CoA ligase [ADP-forming] subunit beta, mitochondrial	IPI00261627	50 kDa				100%		90%			100%		
42	IPI:IPI00268308.5 Tax_Id=10090 Gene_Symbol=Prss46 1700112C13Rik protein	IPI00268308	34 kDa				89%	100%	100%				100%	92%
43	IPI:IPI00131204.1 Tax_Id=10090 Gene_Symbol=Ugp2 Isoform 1 of UTP--glucose-1-phosphate uridylyltransferase	IPI00131204 (+1)	57 kDa			100%					100%			
44	IPI:IPI00874850.1 Tax_Id=10090 Gene_Symbol=Car4 Carbonic anhydrase 4	IPI00874850	34 kDa					100%	100%				100%	100%
45	IPI:IPI00130280.1 Tax_Id=10090 Gene_Symbol=Atp5a1 ATP synthase subunit alpha, mitochondrial	IPI00130280	60 kDa			100%					100%			
46	IPI:IPI00379245.2 Tax_Id=10090 Gene_Symbol=Gnpda1 Glucosamine-6-phosphate isomerase 1	IPI00379245 (+1)	33 kDa					100%	100%				100%	92%
47	IPI:IPI00117705.1 Tax_Id=10090 Gene_Symbol=Ddost Dolichyl-diphosphooligosaccharide--protein glycosyltransferase 48 kDa subunit	IPI00117705 (+1)	49 kDa				100%					100%		
48	IPI:IPI00112876.2 Tax_Id=10090 Gene_Symbol=Prss52 Testicular-specific serine protease 3	IPI00112876 (+1)	36 kDa				100%	100%	90%			100%	100%	100%
49	IPI:IPI00118930.1 Tax_Id=10090 Gene_Symbol=Napa Alpha-soluble NSF attachment protein	IPI00118930	33 kDa						100%					100%
50	IPI:IPI00330862.5 Tax_Id=10090 Gene_Symbol=LOC100044177;Ezr Ezrin	IPI00330862	69 kDa			100%					100%			
51	IPI:IPI00116331.1 Tax_Id=10090 Gene_Symbol=Sgta Isoform 1 of Small glutamine-rich tetratricopeptide repeat-containing protein alpha	IPI00116331 (+1)	34 kDa					100%					100%	
52	IPI:IPI00307837.6 Tax_Id=10090 Gene_Symbol=Eef1a1 Elongation factor 1-alpha 1	IPI00307837	50 kDa				100%	100%	90%			100%	100%	92%
53	IPI:IPI00221402.7 Tax_Id=10090 Gene_Symbol=Aldoa Fructose-bisphosphate aldolase A	IPI00221402 (+1)	39 kDa	TRUE			100%		90%			100%		
54	IPI:IPI00313475.1 Tax_Id=10090 Gene_Symbol=Atp5c1 ATP synthase subunit gamma, mitochondrial	IPI00313475 (+2)	33 kDa						100%					100%
55	IPI:IPI00118279.1 Tax_Id=10090 Gene_Symbol=Slc16a7 Monocarboxylate transporter 2	IPI00118279	53 kDa		100%	99%	100%	100%		91%	76%	90%	100%	
56	IPI:IPI00355382.2 Tax_Id=10090 Gene_Symbol=Wars Isoform 2 of Tryptophanyl-tRNA synthetase, cytoplasmic	IPI00355382	54 kDa			100%	89%				100%	90%		
57	IPI:IPI00116281.3 Tax_Id=10090 Gene_Symbol=Cct6a T-complex protein 1 subunit zeta	IPI00116281	58 kDa			100%		100%			100%		100%	
58	IPI:IPI00122522.1 Tax_Id=10090 Gene_Symbol=Ggt1 Gamma-glutamyltranspeptidase 1	IPI00122522 (+1)	62 kDa			100%					100%			
59	IPI:IPI00127841.3 Tax_Id=10090 Gene_Symbol=Slc25a5 ADP/ATP translocase 2	IPI00127841	33 kDa	TRUE				89%	100%					100%
60	IPI:IPI00122743.1 Tax_Id=10090 Gene_Symbol=Dars Aspartyl-tRNA synthetase, cytoplasmic	IPI00122743 (+1)	57 kDa			100%					100%			
61	IPI:IPI00118594.1 Tax_Id=10090 Gene_Symbol=Pdha2 Pyruvate dehydrogenase E1 component subunit alpha, testis-specific form, mitochondrial	IPI00118594	43 kDa				100%					100%		
62	IPI:IPI00329890.1 Tax_Id=10090 Gene_Symbol=BC049730 cDNA sequence BC049730	IPI00329890	27 kDa					100%	100%				100%	100%
63	IPI:IPI00116222.1 Tax_Id=10090 Gene_Symbol=Hibadh 3-hydroxyisobutyrate dehydrogenase, mitochondrial	IPI00116222	35 kDa						100%					100%
64	IPI:IPI00330480.1 Tax_Id=10090 Gene_Symbol=- 35 kDa protein	IPI00330480	35 kDa	TRUE	100%	83%	99%	89%		100%			89%	99%
65	IPI:IPI00223757.4 Tax_Id=10090 Gene_Symbol=Akr1b3 Aldose reductase	IPI00223757	36 kDa	TRUE				100%					100%	
66	IPI:IPI00112719.1 Tax_Id=10090 Gene_Symbol=Alad Delta-aminolevulinic acid dehydratase	IPI00112719	36 kDa					100%					100%	
67	IPI:IPI00753038.1 Tax_Id=10090 Gene_Symbol=Sord Sorbitol dehydrogenase	IPI00753038 (+1)	38 kDa			83%	100%	100%				100%	100%	
68	IPI:IPI00114209.1 Tax_Id=10090 Gene_Symbol=Glud1 Glutamate dehydrogenase 1, mitochondrial	IPI00114209	61 kDa			100%					100%			
69	IPI:IPI00132475.2 Tax_Id=10090 Gene_Symbol=Lman1 Putative uncharacterized protein	IPI00132475	61 kDa			100%					100%			
70	IPI:IPI00122344.1 Tax_Id=10090 Gene_Symbol=Cth Cystathionine gamma-lyase	IPI00122344	44 kDa				100%					100%		
71	IPI:IPI00469268.5 Tax_Id=10090 Gene_Symbol=Cct8 T-complex protein 1 subunit theta	IPI00469268	60 kDa			100%					100%			
72	IPI:IPI00114801.2 Tax_Id=10090 Gene_Symbol=Inpp1 Inositol polyphosphate 1-phosphatase	IPI00114801	43 kDa				100%					100%		
73	IPI:IPI00131887.3 Tax_Id=10090 Gene_Symbol=Ptgr1 Prostaglandin reductase 1	IPI00131887	36 kDa					100%					100%	73%
74	IPI:IPI00114402.2 Tax_Id=10090 Gene_Symbol=Spam1 Hyaluronidase PH-20	IPI00114402	58 kDa			100%	100%				100%	90%		
75	IPI:IPI00134521.1 Tax_Id=10090 Gene_Symbol=Gapdhs Glyceraldehyde-3-phosphate dehydrogenase, testis-specific	IPI00134521	48 kDa	TRUE		100%					100%			
76	IPI:IPI00113869.1 Tax_Id=10090 Gene_Symbol=Bsg Isoform 2 of Basigin	IPI00113869 (+2)	30 kDa				89%	89%	100%					100%
77	IPI:IPI00411102.3 Tax_Id=10090 Gene_Symbol=Prps2 Ribose-phosphate pyrophosphokinase 2	IPI00411102	35 kDa	TRUE				100%					100%	
78	IPI:IPI00154054.1 Tax_Id=10090 Gene_Symbol=Acat1 Acetyl-CoA acetyltransferase, mitochondrial	IPI00154054	45 kDa				100%					100%		
79	IPI:IPI00123862.1 Tax_Id=10090 Gene_Symbol=Ppp1cc Isoform Gamma-1 of Serine/threonine-protein phosphatase PP1-gamma catalytic subunit	IPI00123862 (+1)	37 kDa	TRUE				100%					100%	
80	IPI:IPI00122548.3 Tax_Id=10090 Gene_Symbol=Vdac3 Voltage-dependent anion channel 3	IPI00122548 (+1)	31 kDa						100%					100%
81	IPI:IPI00266752.2 Tax_Id=10090 Gene_Symbol=Cpne3 Copine-3	IPI00266752	60 kDa			100%					100%			
82	IPI:IPI00314748.7 Tax_Id=10090 Gene_Symbol=Wdr1 WD repeat-containing protein 1	IPI00314748	66 kDa			100%					100%			
83	IPI:IPI00228385.5 Tax_Id=10090 Gene_Symbol=G6pdx Glucose-6-phosphate 1-dehydrogenase X	IPI00228385	59 kDa	TRUE		100%					100%			
84	IPI:IPI00323465.3 Tax_Id=10090 Gene_Symbol=Amdhd2 Putative N-acetylglucosamine-6-phosphate deacetylase	IPI00323465	44 kDa				100%					100%		
85	IPI:IPI00626790.3 Tax_Id=10090 Gene_Symbol=Glul Glutamine synthetase	IPI00626790	42 kDa				100%					100%		
86	IPI:IPI00115275.3 Tax_Id=10090 Gene_Symbol=Anxa13 Annexin A13	IPI00115275	36 kDa						100%					100%
87	IPI:IPI00458922.1 Tax_Id=10090 Gene_Symbol=Atp6v1b1 Putative uncharacterized protein	IPI00458922	57 kDa	TRUE		100%					100%			
88	IPI:IPI00459033.2 Tax_Id=10090 Gene_Symbol=Dnajc3 DnaJ homolog subfamily C member 3	IPI00459033	57 kDa			100%		89%			100%			
89	IPI:IPI00125091.1 Tax_Id=10090 Gene_Symbol=Lasp1 LIM and SH3 domain protein 1	IPI00125091 (+1)	30 kDa					100%					100%	
90	IPI:IPI00133317.1 Tax_Id=10090 Gene_Symbol=Stxbp2 Syntaxin-binding protein 2	IPI00133317 (+1)	66 kDa			100%					100%			
91	IPI:IPI00312018.6 Tax_Id=10090 Gene_Symbol=Mlec Malectin	IPI00312018	32 kDa						100%					100%
92	IPI:IPI00122212.2 Tax_Id=10090 Gene_Symbol=Aldh1a2 Retinal dehydrogenase 2	IPI00122212	57 kDa			100%					100%			
93	IPI:IPI00331436.4 Tax_Id=10090 Gene_Symbol=Lap3 Isoform 1 of Cytosol aminopeptidase	IPI00331436 (+1)	56 kDa			100%					100%			
94	IPI:IPI00331041.2 Tax_Id=10090 Gene_Symbol=Fhl4 four and a half LIM domains 4	IPI00331041	32 kDa	TRUE					100%					100%
95	IPI:IPI00314950.2 Tax_Id=10090 Gene_Symbol=Rplp0 60S acidic ribosomal protein P0	IPI00314950 (+1)	34 kDa					100%					100%	
96	IPI:IPI00120716.3 Tax_Id=10090 Gene_Symbol=Gnb1 Guanine nucleotide-binding protein G(I)/G(S)/G(T) subunit beta-1	IPI00120716	37 kDa					100%					100%	
97	IPI:IPI00953754.1 Tax_Id=10090 Gene_Symbol=Prps1l1 phosphoribosyl pyrophosphate synthetase 1-like 1	IPI00953754	35 kDa	TRUE				100%					98%	
98	IPI:IPI00136744.1 Tax_Id=10090 Gene_Symbol=Igbp1b Immunoglobulin-binding protein 1b	IPI00136744	39 kDa				100%					88%		

99	IPI:IPI00338561.4 Tax_Id=10090 Gene_Symbol=Sil1 Nucleotide exchange factor SIL1	IPI00338561	52 kDa		100%					100%			
100	IPI:IPI00110224.1 Tax_Id=10090 Gene_Symbol=Hsd17b11 Isoform 1 of Estradiol 17-beta-dehydrogenase 11	IPI00110224 (+1)	33 kDa					100%					100%
101	IPI:IPI00230139.5 Tax_Id=10090 Gene_Symbol=Fkbp4 FK506-binding protein 4	IPI00230139	52 kDa		100%					100%			
102	IPI:IPI00411056.1 Tax_Id=10090 Gene_Symbol=Atp8b3 SAPLT	IPI00411056 (+1)	152 kDa	100%					91%				
103	IPI:IPI00283264.2 Tax_Id=10090 Gene_Symbol=Arrdc5 Arrestin domain-containing protein 5	IPI00283264	37 kDa				89%					100%	
104	IPI:IPI00129319.3 Tax_Id=10090 Gene_Symbol=Ppp1r7 Protein phosphatase 1 regulatory subunit 7	IPI00129319	41 kDa			100%					100%		
105	IPI:IPI00475154.1 Tax_Id=10090 Gene_Symbol=Rpn2 Dolichyl-diphosphooligosaccharide--protein glycosyltransferase subunit 2	IPI00475154 (+2)	69 kDa		100%					76%			

Table S2. Proteins with known or potential enzymatic activities enriched in murine cytoplasmic droplets

Gene symbol	Enzymatic activity	Function in sperm and in male reproduction	Known inhibitors
Acat1	metal ion binding	NA	None
Ace	regulation of blood pressure;dipeptidyl-carboxypeptidase activity	Male fertilization; zona pellucida binding	Ramipril;Captopril; lisinopril;Fosinopril; Enalapril; Quinapril; Cilazapril
Akr1b3	alditol:NADP+ 1-oxidoreductase activity, glyceraldehyde oxidoreductase activity	NA	GP-1447 (3-[(4,5,7-trifluorobenzothiazol-2-yl)methyl]-5-methylphenyl acetic acid); WJ-38;β-glucogallin
Akr1b7	detoxifying processes; aldehyde reductase activity	No phenotype for male	None
Alad	porphobilinogen synthase activity; identical protein binding	biochemical parameters of semen quality	None
Aldh1a1	retinal dehydrogenase activity; 3-chloroallyl aldehyde dehydrogenase activity	NA	None
Aldh1a2	3-chloroallyl aldehyde dehydrogenase activity	a promising target for the development of a reversible, nonhormonal male contraceptive; testicular retinoic acid biosynthesis	bisdichloroacetyldiamine WIN 18,446
Aldoa	glycolysis for fructose-bisphosphate aldolase activity	support sperm motility	None
Amdhd2	N-acetylglucosamine-6-phosphate deacetylase activity;hydrolase activity	NA	None
Atp1b3	sodium:potassium-exchanging ATPase activity	NA	None
Atp5a1	ATP binding	NA	None
Atp5c1	hydrogen ion transporting ATPase activity, rotational mechanism	NA	None
Atp6v1b1	hydrogen ion transmembrane transporter activity	major contributor to luminal acidification	Bafilomycin A1
Atp6v1b2	hydrogen ion transporting ATPase activity, rotational mechanism	establish an acidic luminal pH in epididymis and vas deferens	None
Atp8b3	ATP binding	Acrosome development and/or in sperm function during fertilization	None
Car4	carbonate dehydratase activity; lyase activity; zinc ion binding	acidification of the epididymal fluid that prevents premature sperm activation and regulation of sperm maturation and motility	acetazolamide (AZ); 2-(4-sulfamoylphenyl-amino)-4,6-dichloro-1,3,5-triazine (TR1); 4-[3-(N,N-dimethylaminopropyl)thioreidophenylsulfonylaminoethyl]benzenesulfonamide (GA15); Sulthiame
Ces3	carboxylesterase activity, fatty-acyl-ethyl-ester synthase activity	NA	None
Cpne3	protein serine/threonine kinase activity	NA	None
Cth	pyridoxal phosphate binding;calmodulin binding	NA	DL-propargyl glycine;DL-propargylglycine
Dars	aspartate-tRNA ligase activity	NA	chloramphenicol analogs
Ddost	dolichyl-diphosphooligosaccharide-protein glycotransferase activity	NA	None
Dnpep	aminopeptidase activity; zinc ion binding; proteolysis	NA	None
Dpep3	metal ion binding;proteolysis	interactions with the Ts4 antigens, including TEX101, in testis	None
Eef1a1	GTP binding	Localization in the midpiece of the sperm tail, invole in PASKIN-dependent phosphorylation	Gamendazole
Fbp1	metal ion binding; AMP binding; catalytic activity	Key substrate cycle enzyme and control of glycolysis in spermatid	adenosine monophosphate (AMP); MB07803
Fkbp4	peptidyl-prolyl cis-trans isomerase activity; copper-dependent protein binding; GTP binding	fertilizing capacity and sperm morphology;	None
G6pd2	glucose-6-phosphate dehydrogenase activity; NADP binding	probably related to X chromosome inactivation during spermatogenesis;	beta-naphthoquinone-4-sulfonic acid; Cu2+;Dehydroepiandrosterone (DHEA)
G6pdx	protein homodimerization activity;carbohydrate binding	NA	None
Gapdhs	glyceraldehyde-3-phosphate dehydrogenase (NAD+) (phosphorylating) activity, NAD binding	sperm motility and male fertility	Koningic acid (heptelidic acid); alpha-Chlorohydrin;ornidazole
Ggt1	gamma-glutamyltransferase activity;	sperm GGT may be a limiting factor for male pronuclear formation in polyspermic oocytes	serine/borate; OU749; acivicin
Glud1	glutamate dehydrogenase [NAD(P)+] activity; positive regulation of insulin secretion; ADP binding	NA	glutamate dehydrogenases
Glul	ATP binding	maintaining an optimal microenvironment for sperm maturation	methionine sulfoximine
Gm3272;LOC100042025;Gm12033;Gapdh;Gm10293;Gm10359;Gm2574;Gm5138	glyceraldehyde-3-phosphate dehydrogenase (phosphorylating) activity	sperm motility	ornidazole; alpha-chlorohydrin; 1-chloro-3-hydroxypropanone.
Gnpda1	glucosamine-6-phosphate deaminase activity	NA	None
Gpi1	glucose-6-phosphate isomerase activity	involved in sperm agglutination	None
Hibadh	NAD binding; pentose-phosphate shunt	NA	None
Hsd17b11	steroid biosynthetic process; estradiol 17-beta-dehydrogenase activity; nucleotide binding	NA	None
Inpp1	metal ion binding; inositol-1,4-bisphosphate 1-phosphatase activity	NA	None

Lap3	metal ion binding;aminopeptidase activity; proteolysis	NA	Bestatin
Ldhal6b	L-lactate dehydrogenase activity, oxidoreductase activity	NA	None
Ldhc	L-lactate dehydrogenase activity; oxidation reduction	required for male fertility and sperm function	sodium oxamate
Oxct2a	3-oxoacid CoA-transferase activity; ketone body catabolic process	testicular haploid germ cell-specific gene	None
P4hb	electron carrier activity, isomerase activity	esponsible for spermatogenesis	testosterone undecanoate; levonorgestrel
Pcyox1	prenylcysteine catabolic process; oxidoreductase activity,	NA	None
Pdha2	pyruvate dehydrogenase (acetyl-transferring) activity; oxidoreductase activity	tyrosine phosphorylated in capacitated spermatozoa and required for sperm hyperactivation and acrosome reaction	bromopyruvate (BP)
Pdia3	protein disulfide isomerase activity; electron carrier activity	play a critical role in gamete fusion;	None
Pgk2	ATP binding; kinase activity	associated with male fertility and sperm function	2-oxo-1,5-bisphosphonopentane
Phgdh	NAD binding	NA	Koningic acid; l-Serine
Ppp1cc	protein serine/threonine phosphatase activity; protein amino acid dephosphorylation; metal ion binding	required for spermiogenesis	Phosphatidic acid (PA)
Ppp3cc	phosphoprotein phosphatase activity; calmodulin binding	regulation of flagellar motility	None
Prps1	protein homodimerization activity; ribose phosphate diphosphokinase activity; ADP binding	NA	aminopyrimidopyrimidine nucleotide 4-amino-8-(beta-D-ribofuranosylamino)pyrimido[5,4-d]pyrimidine[-5' -monophosphate (APP-MP)
Prps1l1	kinase activity; metal ion binding	NA	None
Prps2	protein homodimerization activity;ribose phosphate diphosphokinase activity; ADP binding	NA	aminopyrimidopyrimidine nucleotide 4-amino-8-(beta-D-ribofuranosylamino)pyrimido[5,4-d]pyrimidine[-5' -monophosphate (APP-MP)
Prss21	serine-type endopeptidase activity; catalytic activity	regulation of epididymal sperm cell maturation and sperm-fertilizing ability	None
Prss46	molecular function unknown	NA	None
Prss52	serine-type endopeptidase activity; proteolysis	NA	None
Psmc6	ATP binding; nucleoside-triphosphatase activity; protein catabolic process	have a role in the remodeling of the outer dense fibers and other tail components during epididymal transit; degrade an ubiquitinated ZP (glyco)protein during fertilization	MG132
Psmd14	metal ion binding; ubiquitin thiolesterase activity	NA	None
Psmd3	regulation of protein catabolic process;enzyme regulator activity	NA	None
Psmd6	unknown	NA	None
Ptgr1	2-alkenal reductase activity;13-prostaglandin reductase activity; 15-oxoprostaglandin 13-oxidase activity	NA	None
Rpn2	dolichyl-diphosphooligosaccharide-protein glycotransferase activity; ribosome binding	NA	None
Sgta	protein heterodimerization activity; protein homodimerization activity; binding	NA	None
Slc25a5	transporter activity; binding	NA	None
Sord	L-iditol 2-dehydrogenase activity; identical protein binding	sperm motility and protein tyrosine phosphorylation	CP-642,931; SDI-711
Spam1	hyaluronoglucosaminidase activity; catalytic activity	function in sperm entry into the cumulus and sperm penetration through the cumulus matrix during fertilization	polyphenols (tannic acid, apigenin and quercetin) ; sodium cromoglycate and sodium auro-thiomalate
Sucla2	ATP binding; succinate-CoA ligase (ADP-forming) activity;	NA	None
Uggt1	UDP-glucose:glycoprotein glucosyltransferase activity; protein amino acid glycosylation	NA	None
Ugp2	metal ion binding; UTP:glucose-1-phosphate uridylyltransferase activity	NA	None
Vdac3	porin activity; nucleotide binding	Function in sperm motility and epididymal axonemes structure	None
Wars	ATP binding; tryptophan-tRNA ligase activity	NA	None

Table S3. Species with epididymal sperm bearing cytoplasmic droplets.

Species	Literatures
Human	(1-4)
Monkey	(5, 6)
Marmoset	(7)
Stallion	(8)
Donkey	(9)
Boar	(10-14)
Bovine	(15, 16)
Goat	(17)
Ram	(18, 19)
Llamas	(20)
Cheetah	(21)
Tiger	(21)
Leopard	(21)
Puma	(21)
Cat	(22)
Wolf	(23)
Dog	(24-28)
African elephant	(29, 30)
Rabbit	(19, 31, 32)
Guinea pig	(33-35)
Hamster	(19, 36, 37)
Rat	(19, 38-40)
Mouse	(18)
Bat	(41)
Macropus	(42)
Trichosurus vulpecula	(43, 44)
Bandicoot	(45)
Echidna	(46)
Platypus	(47)
Ostrich	(48)
Hagfish	(49)

References cited in Table S3:

1. Fetic S, Yeung CH, Sonntag B, Nieschlag E, & Cooper TG (2006) Relationship of cytoplasmic droplets to motility, migration in mucus, and volume regulation of human spermatozoa. (Translated from eng) *J Androl* 27(2):294-301 (in eng).
2. Weng SL*, et al.* (2002) Caspase activity and apoptotic markers in ejaculated human sperm. (Translated from eng) *Mol Hum Reprod* 8(11):984-991 (in eng).
3. Naaby-Hansen S*, et al.* (2001) Co-localization of the inositol 1,4,5-trisphosphate receptor and calreticulin in the equatorial segment and in membrane bounded vesicles in the cytoplasmic droplet of human spermatozoa. (Translated from eng) *Mol Hum Reprod* 7(10):923-933 (in eng).
4. Panidis D, Rousso D, Vlassis G, & Kalogeropoulos A (1990) Coexistence of spermatozoa morphological abnormalities in the semen of subfertile men with varicocele. (Translated from eng) *Eur J Obstet Gynecol Reprod Biol* 37(2):175-181 (in eng).
5. Mahony MC*, et al.* (1993) Functional and morphological features of spermatozoa microaspirated from the epididymal regions of cynomolgus monkeys (Macaca fascicularis). (Translated from eng) *Biol Reprod* 48(3):613-620 (in eng).
6. Ramakrishnan PR, Kaur J, & Rajalakshmi M (1990) Effect of non-aromatizable androgens on testicular and accessory gland functions in rhesus monkeys (Macaca mulatta). (Translated from eng) *J Reprod Fertil* 89(1):69-76 (in eng).
7. Moore HD, Hartman TD, & Holt WV (1984) The structure and epididymal maturation of the spermatozoon of the common marmoset (Callithrix jacchus). (Translated from eng) *J Anat* 138 (Pt 2):227-235 (in eng).
8. Huszar G*, et al.* (1998) Cytoplasmic extrusion and the switch from creatine kinase B to M isoform are completed by the commencement of epididymal transport in human and stallion spermatozoa. (Translated from eng) *J Androl* 19(1):11-20 (in eng).
9. Gloria A, Contri A, De Amicis I, Robbe D, & Carluccio A (2011) Differences between epididymal and ejaculated sperm characteristics in donkey. (Translated from eng) *Anim Reprod Sci* 128(1-4):117-122 (in eng).
10. Lovercamp KW*, et al.* (2007) High resolution light microscopic evaluation of boar semen quality sperm cytoplasmic droplet retention in relationship with boar fertility parameters. (Translated from eng) *Arch Androl* 53(4):219-228 (in eng).
11. Kuster CE, Hess RA, & Althouse GC (2004) Immunofluorescence reveals ubiquitination of retained distal cytoplasmic droplets on ejaculated porcine spermatozoa. (Translated from eng) *J Androl* 25(3):340-347 (in eng).
12. Holt WV (1982) Epididymal origin of a coiled-tail sperm defect in a boar. (Translated from eng) *J Reprod Fertil* 64(2):485-489 (in eng).
13. Kaplan M, Russell LD, Peterson RN, & Martan J (1984) Boar sperm cytoplasmic droplets: their ultrastructure, their numbers in the epididymis and at ejaculation and their removal during isolation of sperm plasma membranes. (Translated from eng) *Tissue Cell* 16(3):455-468 (in eng).
14. Larsen RE, Shope RE, Jr., Leman AD, & Kurtz HJ (1980) Semen changes in boars after experimental infection with pseudorabies virus. (Translated from eng) *Am J Vet Res* 41(5):733-739 (in eng).

15. Reyes R, Vazquez ML, & Delgado NM (2004) Detection and bioimaging of nitric oxide in bovine oocytes and sperm cells. (Translated from eng) *Arch Androl* 50(4):303-309 (in eng).
16. Garbers DL, Wakabayashi T, & Reed PW (1970) Enzyme profile of the cytoplasmic droplet from bovine epididymal spermatozoa. (Translated from eng) *Biol Reprod* 3(3):327-337 (in eng).
17. Jindal SK & Panda JN (1980) Maturation changes of goat spermatozoa during transit throught the epididymis. (Translated from eng) *Andrologia* 12(4):328-331 (in eng).
18. Schlegel RA*, et al.* (1986) Changes in the organization of the lipid bilayer of the plasma membrane during spermatogenesis and epididymal maturation. (Translated from eng) *Biol Reprod* 34(2):379-391 (in eng).
19. Moniem KA & Glover TD (1972) Alkaline phosphatase in the cytoplasmic droplet of mammalian spermatozoa. (Translated from eng) *J Reprod Fertil* 29(1):65-69 (in eng).
20. Delhon G & von Lawzewitsch I (1994) Ductus epididymidis compartments and morphology of epididymal spermatozoa in llamas. (Translated from eng) *Anat Histol Embryol* 23(3):217-225 (in eng).
21. Wildt DE*, et al.* (1988) A comparative analysis of ejaculate and hormonal characteristics of the captive male cheetah, tiger, leopard, and puma. (Translated from eng) *Biol Reprod* 38(2):245-255 (in eng).
22. Axner E, Linde-Forsberg C, & Einarsson S (1999) Morphology and motility of spermatozoa from different regions of the epididymal duct in the domestic cat. (Translated from eng) *Theriogenology* 52(5):767-778 (in eng).
23. Goodrowe KL*, et al.* (1998) Characteristics of fresh and frozen-thawed red wolf (Canis rufus) spermatozoa. (Translated from eng) *Anim Reprod Sci* 53(1-4):299-308 (in eng).
24. Pena AI, Barrio M, Becerra JJ, Quintela LA, & Herradon PG (2007) Infertility in a dog due to proximal cytoplasmic droplets in the ejaculate: investigation of the significance for sperm functionality in vitro. (Translated from eng) *Reprod Domest Anim* 42(5):471-478 (in eng).
25. Veeramachaneni DN, Smith MO, & Ellinwood NM (1998) Deficiency of fucosidase results in acrosomal dysgenesis and impaired sperm maturation. (Translated from eng) *J Androl* 19(4):444-449 (in eng).
26. Vanderhaeghen P, Schurmans S, Vassart G, & Parmentier M (1993) Olfactory receptors are displayed on dog mature sperm cells. (Translated from eng) *J Cell Biol* 123(6 Pt 1):1441-1452 (in eng).
27. Bains HK, Pabst MA, & Bawa SR (1993) Changes in the lectin binding sites on the testicular, epididymal, vas, and ejaculated spermatozoon surface of dog. (Translated from eng) *Andrologia* 25(1):19-24 (in eng).
28. Hrudka F (1983) Acrosomo-nuclear syndrome in canine sperm. (Translated from eng) *Andrologia* 15(4):310-321 (in eng).
29. Howard JG, Bush M, de Vos V, & Wildt DE (1984) Electroejaculation, semen characteristics and serum testosterone concentrations of free-ranging african elephants (Loxodonta africana). (Translated from eng) *J Reprod Fertil* 72(1):187-195 (in eng).

30. Jones RC (1980) Luminal composition and maturation of spermatozoa in the genital ducts of the African elephant (Loxodonta africana). (Translated from eng) *J Reprod Fertil* 60(1):87-93 (in eng).
31. Perez-Sanchez F, Tablado L, & Soler C (1997) Sperm morphological abnormalities appearing in the male rabbit reproductive tract. (Translated from eng) *Theriogenology* 47(4):893-901 (in eng).
32. Bavdek S & Glover TD (1970) Alkaline phosphatase in the cytoplasmic droplet of rabbit spermatozoa. (Translated from eng) *J Reprod Fertil* 22(2):371-373 (in eng).
33. Suzuki-Toyota F, Ito C, Maekawa M, Toyama Y, & Toshimori K (2010) Adhesion between plasma membrane and mitochondria with linking filaments in relation to migration of cytoplasmic droplet during epididymal maturation in guinea pig spermatozoa. (Translated from eng) *Cell Tissue Res* 341(3):429-440 (in eng).
34. Shi QX & Friend DS (1985) Effect of gossypol acetate on guinea pig epididymal spermatozoa in vivo and their susceptibility to capacitation in vitro. (Translated from eng) *J Androl* 6(1):45-52 (in eng).
35. Mollenhauer HH & Morre DJ (1981) Selective staining of dictyosome-like structures (DLS) from spermatocytes of the guinea pig using phosphotungstic acid at low pH. (Translated from eng) *Eur J Cell Biol* 25(2):340-345 (in eng).
36. Gottlieb W & Meizel S (1987) Biochemical studies of metalloendoprotease activity in the spermatozoa of three mammalian species. (Translated from eng) *J Androl* 8(1):14-24 (in eng).
37. Cummins JM (1976) Effects of epididymal occlusion on sperm maturation in the hamster. (Translated from eng) *J Exp Zool* 197(2):183-190 (in eng).
38. Oko R, Hermo L, Chan PT, Fazel A, & Bergeron JJ (1993) The cytoplasmic droplet of rat epididymal spermatozoa contains saccular elements with Golgi characteristics. (Translated from eng) *J Cell Biol* 123(4):809-821 (in eng).
39. Hermo L, Dworkin J, & Oko R (1988) Role of epithelial clear cells of the rat epididymis in the disposal of the contents of cytoplasmic droplets detached from spermatozoa. (Translated from eng) *Am J Anat* 183(2):107-124 (in eng).
40. Roberts ML, Scouten WH, & Nyquist SE (1976) Isolation and characterization of the cytoplasmic droplet in the rat. (Translated from eng) *Biol Reprod* 14(4):421-424 (in eng).
41. Cervantes MI*, et al.* (2008) Spermatozoa epididymal maturation in the Mexican big-eared bat (Corynorhinus mexicanus). (Translated from eng) *Syst Biol Reprod Med* 54(4-5):196-204 (in eng).
42. Jones RC, Hinds LA, & Tyndale-Biscoe CH (1984) Ultrastructure of the epididymis of the tammar, Macropus eugenii, and its relationship to sperm maturation. (Translated from eng) *Cell Tissue Res* 237(3):525-535 (in eng).
43. Temple-Smith PD (1984) Phagocytosis of sperm cytoplasmic droplets by a specialized region in the epididymis of the brushtailed possum, Trichosurus vulpecula. (Translated from eng) *Biol Reprod* 30(3):707-720 (in eng).
44. Temple-Smith PD & Bedford JM (1976) The features of sperm maturation in the epididymis of a marsupial, the brushtailed possum Trichosurus vulpecula. (Translated from eng) *Am J Anat* 147(4):471-499 (in eng).

45. Taggart DA, Leigh CM, & Breed WG (1995) Ultrastructure and motility of spermatozoa in the male reproductive tract of perameloid marsupials. (Translated from eng) *Reprod Fertil Dev* 7(5):1141-1156 (in eng).
46. Djakiew D & Jones RC (1983) Sperm maturation, fluid transport, and secretion and absorption of protein in the epididymis of the echidna, Tachyglossus aculeatus. (Translated from eng) *J Reprod Fertil* 68(2):445-456 (in eng).
47. Lin M & Jones RC (2000) Spermiogenesis and spermiation in a monotreme mammal, the platypus, Ornithorhynchus anatinus. (Translated from eng) *J Anat* 196 (Pt 2):217-232 (in eng).
48. Aire TA & Soley JT (2000) The surface features of the epithelial lining of the ducts of the epididymis of the ostrich (Struthio camelus). (Translated from eng) *Anat Histol Embryol* 29(2):119-126 (in eng).
49. Morisawa S (2005) Spermiogenesis in the Hagfish Eptatretus burgeri (Agnatha). (Translated from eng) *Biol Bull* 209(3):204-214 (in eng).

Supporting Videos:

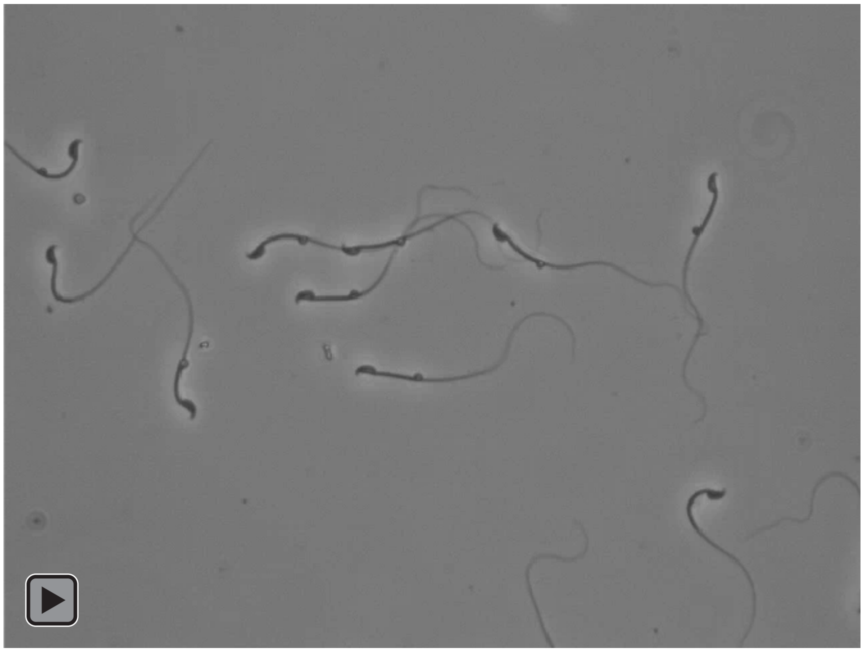

Supporting Video S1. CD-bearing murine epididymal spermatozoa displayed vigorous motility after activation in HTF-HEPES medium at 37°C for 30 minutes.

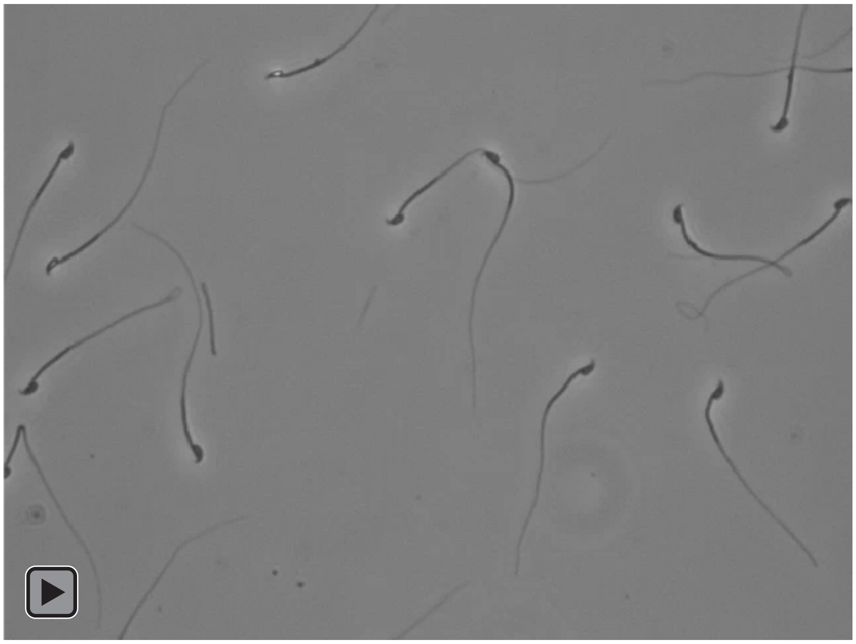

Supporting Video S2. "CD-less" murine epididymal spermatozoa developed no motility after activation in HTF-HEPES medium at 37°C for 30 minutes.
